# Synthesis of trifluoromethyl ketones by nucleophilic trifluoromethylation of esters under a fluoroform/KHMDS/triglyme system

**DOI:** 10.3762/bjoc.17.39

**Published:** 2021-02-12

**Authors:** Yamato Fujihira, Yumeng Liang, Makoto Ono, Kazuki Hirano, Takumi Kagawa, Norio Shibata

**Affiliations:** 1 Department of Life Science and Applied Chemistry, Nagoya Institute of Technology, Gokiso, Showa-ku, Nagoya 466-5888, Japan; 2 Department of Nanopharmaceutical Sciences, Nagoya Institute of Technology, Gokiso, Showa-ku, Nagoya 466-5888, Japan; 3 Tosoh Finechem Corporation, 4988, Kaiseicho, Shunan, 746-0006, Japan; 4 Institute of Advanced Fluorine-Containing Materials, Zhejiang Normal University, 688 Yingbin Avenue, 321004 Jinhua, China

**Keywords:** fluoroform, greenhouse gas, HFC-23, trifluoromethyl ketones, trifluoromethylation

## Abstract

A straightforward method that enables the formation of biologically attractive trifluoromethyl ketones from readily available methyl esters using the potent greenhouse gas fluoroform (HCF_3_, HFC-23) was developed. The combination of fluoroform and KHMDS in triglyme at −40 °C was effective for this transformation, with good yields as high as 92%. Substrate scope of the trifluoromethylation procedure was explored for aromatic, aliphatic, and conjugated methyl esters. This study presents a straightforward trifluoromethylation process of various methyl esters that convert well to the corresponding trifluoromethyl ketones. The tolerance of various pharmacophores under the reaction conditions was also explored.

## Introduction

**Scheme 1:**
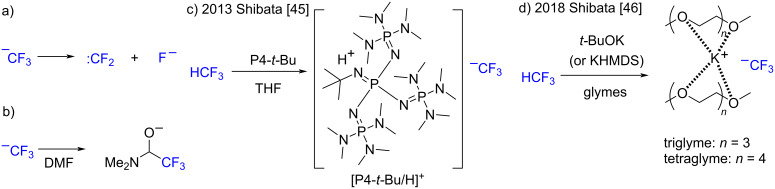
Chemistry of the CF_3_ anion generated from HCF_3_. a) Decomposition of the trifluoromethyl anion to difluorocarbene and fluoride. b) A hemiaminaloate adduct of CF_3_ anion to DMF. c) Formation of the [P4-*t*-Bu]H^+^ CF_3_ anion salt. d) Encapsulation of K^+^ by glymes. Transformation of esters to trifluoromethyl ketones.

In recent decades, organofluorine molecules have received widespread attention in the field of medicinal chemistry [1–4]. The introduction of fluorine(s) into organic molecules usually leads to significant changes in the chemical and physicochemical properties of the original compounds [5–6]. Hence, the fluorination and related fluoro-functionalization of drug candidates are powerful strategies in drug design to appropriately bias their biological properties, bioavailability, and ADME [7–8]. While tremendous methodologies have been developed for the synthesis of organofluorine compounds [9–10], many of the laboratory methods are not always suitable for industrial production in terms of their synthetic complexity, handling, and cost of target compounds [11–15]. Thus, the development of low-cost and straightforward chemical synthetic technologies, including fluorination and trifluoromethylation, are matters of considerable importance to pharmaceutical and agrochemical industries. Fluoroform (HCF_3_, HFC-23) is an industrial byproduct of polytetrafluoroethylene synthesis and has become an ideal, economical feedstock for trifluoromethyl (CF_3_) compounds. Rather than decomposing CF_3_ compounds, it would be better to maximize the efficiency of their use [16–18]. However, taming HCF_3_ as a trifluoromethylation agent is a challenge in organic chemistry [19–28], although recent rapid progress in the chemistry of HCF_3_ by Grushin (for CuCF_3_) [29–37], Prakash (for KCF_3_) [38], and others [39–44], including our group [45–50], has dramatically improved the prospects. One of the problems facing the treatment of HCF_3_ for nucleophilic trifluoromethylation reactions is the low stability of the directly generated CF_3_ anion (CF_3_^−^) for decomposing to difluorocarbene (:CF_2_) and fluoride (F^−^) (Scheme 1a). Due to the formation of highly stable fluoride salts (MF), the breakdown of CF_3_^−^ into difluorocarbene in the presence of alkali (M^+^) and other metal cations is favored. In earlier studies, the solvent *N,N*-dimethylformamide (DMF), was essential for nucleophilic trifluoromethylation by HCF_3_ since DMF acts as a CF_3_ anion reservoir that is used as a hemiaminaloate adduct [Me_2_NCH(O)CF_3_]- (Scheme 1b) [19–28]. Although the taming CF_3_ anion had been an elusive problem for decades, it has been dramatically progressed in recent years by the substantial works by Grushin [51–53] and Prakash [54]. Our group reported novel DMF-free systems for the nucleophilic trifluoromethylation reaction using HCF_3_, including the phosphazene base P4-*t*-Bu (P4-*t*-Bu), in 2013 (Scheme 1c) [45] and a potassium *tert*-butoxide (*t*-BuOK) or potassium hexamethyldisilazide (KHMDS)/glyme combination in 2018 (Scheme 1d) [46]. The success of our DMF-free systems lies in the generation of sterically demanding cationic species, [P4-*t*-Bu]H^+^ or glyme capsulized K^+^, resulting in the stabilization of CF_3_^−^ from HCF_3_ by ion separation. The sterically demanding [P4-*t*-Bu]H^+^ or encapsulation of K^+^ by glymes effectively inhibits the contact of CF_3_^−^ to K^+^, preventing decomposition into CF_2_ and KF. The isolated CF_3_ is rather naked with a highly nucleophilic character, which is suitable for nucleophilic trifluoromethylation reactions. The K^+^ and glyme combination is particularly useful for the nucleophilic trifluoromethylation of carbonyl compounds to trifluoromethyl carbinols because it does not require any expensive reagents nor very low-temperature conditions. Although the reaction has a broad substrate scope of embracing ketones, chalcones and aldehydes, the transformation of esters to trifluoromethyl ketones by this protocol was never examined [46].

**Figure 1:**
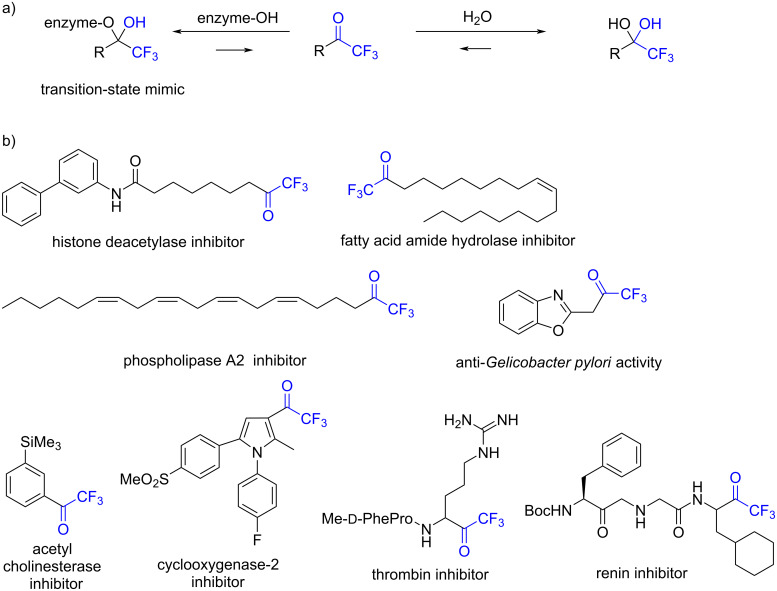
Trifluoromethyl ketones. a) Hydrolysis of trifluoromethyl ketones. b) Selected examples of biologically active trifluoromethyl ketones.

Trifluoromethyl ketones (TFMKs) are valuable fluorine-containing synthetic targets of bioactive compounds [55–56] that behave as mimics of the tetrahedral transition-state intermediate of enzymatic hydrolysis of esters and amides by stabilizing their hydrates (Figure 1a) [57]. In fact, the TFMK moiety is a proven effective metal chelator in various enzyme inhibitors (Figure 1b) [58–65].

**Scheme 2:**
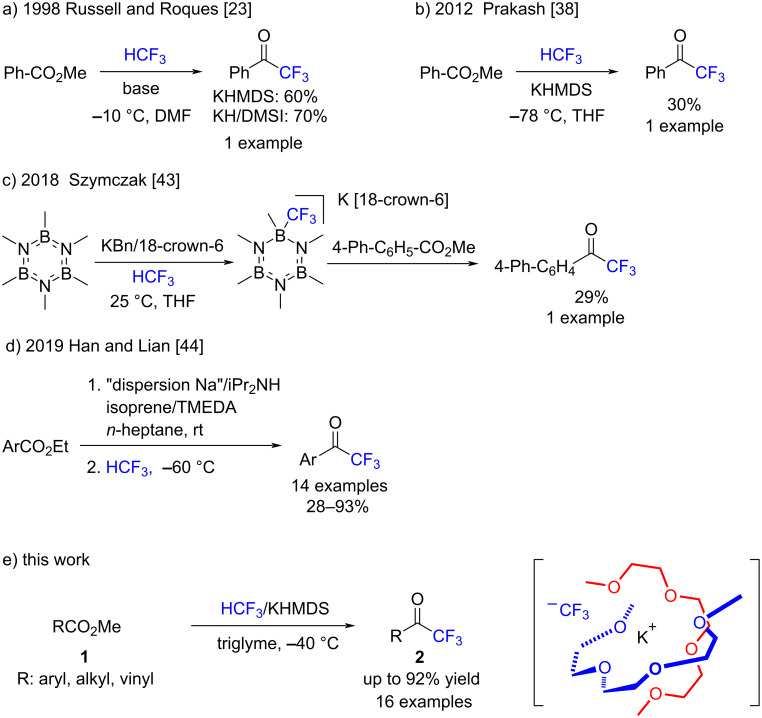
Trifluoromethylation of esters by HCF_3_ by a) Russell and Roques (1998), b) Prakash and co-workers (2012), c) Szymczak and co-workers (2018), d) Han, Lian and co-workers (2019), and e) our group in this work.

Several useful methods exist for preparing trifluoromethyl ketones [66–67], such as the direct trifluoromethylation of esters by the Ruppert–Prakash reagent (Me_3_SiCF_3_) [68–71], but the use of HCF_3_ for this transformation reaction is still limited. In 1998, Russel and Roques examined the transformation of methyl benzoate to trifluoromethyl phenyl ketone with HCF_3_ in the presence of KHMDS or KH/DMSO in DMF, but the method required DMF and only a single example was indicated (Scheme 2a) [23]. Prakash and co-workers showed the first example of the DMF-free preparation of trifluoromethyl phenyl ketone with HCF_3_ in the presence of KHMDS in THF, but they did not examined the scope of the reaction (Scheme 2b) [38]. In 2018, Szymczak and co-workers showed a single example of the preparation of phenyl trifluoromethyl ketone using HCF_3_-derived borazine CF_3_^–^ in 29% yield (Scheme 2c) [43]. Very recently, Han, Lian, and co-workers reported that a protocol using diisopropylaminosodium (NaDA) was useful for the trifluoromethylation of esters to trifluoromethyl ketones with HCF_3_ at −60 °C (Scheme 2d) [44]. However, the preparation of NaDA was rather complicated and required pre-mixing of diisopropylamine, tetramethylethylenediamine (TMEDA), isoprene, and even more tedious “dispersion sodium” in *n*-heptane at 25 °C for 4 h, before the reaction of esters with HCF_3_ at −60 °C. We herein extend our glyme strategy [50] shown in Scheme 1d, the HCF_3_/KHMDS/triglyme system, for the synthesis of trifluoromethyl ketones from esters (Scheme 2e). The combination of HCF_3_ and KHMDS in triglyme at −40 °C was found to be effective for this transformation, with good yields as high as 92%. The substrate scope of the trifluoromethylation procedure was explored for aromatic, aliphatic, and conjugated methyl esters. This study presents a straightforward trifluoromethylation process of various methyl esters that convert well to the corresponding trifluoromethyl ketones. The tolerance of various pharmacophores under the reaction conditions was also explored.

## Results and Discussion

We first examined the trifluoromethylation reaction of methyl 2-naphthoate (**1a**) as a model substrate for HCF_3_ to optimize the reaction conditions (Table 1). Following our glymes strategy, we initially used *t-*BuOK as the base in triglyme, and the desired trifluoromethyl ketone **2a** was obtained in 29% yield (Table 1, entry 1). We next carried out the reaction in other solvents, THF (**2a**, 5%, Table 1, entry 2) and toluene (**2a**, 0%, Table 1, entry 3), and confirmed the advantage of the triglyme that was used (Table 1, entries 1–3). Increasing the amount of *t-*BuOK to 4.0 equiv did not improve the yield (25%) of **2a** (Table 1, entry 4). When we used KHMDS to replace *t-*BuOK, the yield of **2a** improved significantly to 57% (Table 1, entry 5). As expected, tetraglyme, instead of triglyme, gave a similar good yield of 59% (Table 1, entry 6), while the transformation decreased significantly when diglyme was used (**2a**, 29%, Table 1, entry 7). Interestingly, when we stopped the reaction after 4 h, the yield increased to 76% (Table 1, entry 8). On this basis, we attempted to reduce the amount of HCF_3_ to 1.1 equiv and found that the yield was not sacrificed, yielding 75% of **2a** (Table 1, entry 9). Other optimized reaction conditions did not improve the yield (see Supporting Information File 1 for an extensive list of reaction conditions, Table S1).

**Table 1:** Optimized reaction conditions for the conversion of **1a** to **2a**.

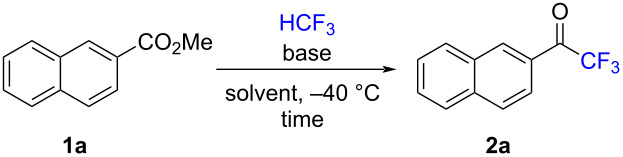				

Entry	Base (equiv)	Solvent	Time	Yield (%)^a^

1	*t-*BuOK (2.0)	triglyme	overnight	29
2	*t-*BuOK (2.0)	THF	overnight	5
3	*t-*BuOK (2.0)	toluene	overnight	0
4	*t-*BuOK (4.0)	triglyme	overnight	25
5	KHMDS (2.0)	triglyme	overnight	57
6	KHMDS (2.0)	tetraglyme	overnight	59
7	KHMDS (2.0)	diglyme	overnight	29
8	KHMDS (2.0)	triglyme	4 h	76
9^b^	KHMDS (2.0)	triglyme	4 h	75 (71)^c^

^a^Determined by ^19^F NMR using a crude mixture with trifluorotoluene as the internal standard. ^b^HCF_3_ was 1.1 equiv. ^c^Isolated yield.

**Scheme 3:**
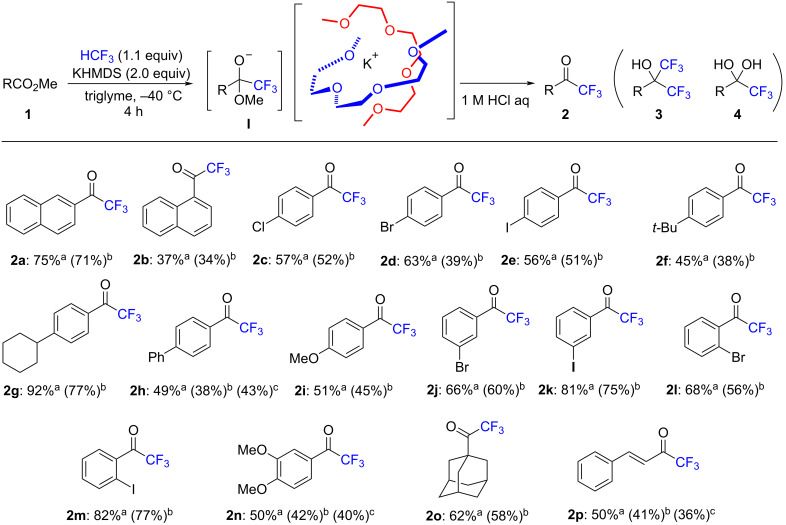
Substrate scope of esters **1** for trifluoromethylation by HCF_3_ under the optimized conditions. ^a^Determined by ^19^F NMR of the crude **2** with trifluorotoluene as an internal standard. ^b^Isolated yield. ^c^Isolated yield of gram-scale reaction by using 1 g of substrate.

We explored the substrate scope of this trifluoromethylation reaction with the optimized conditions in hand (entry 9, Table 1). Various carboxylic esters were investigated in the presence of 1.1 equiv of HCF_3_ and two equiv of KHMDS (Scheme 3). Methyl 2-naphthoate (**1a**) gave **2a** in 75% yield, but sterically demanding methyl 1-naphthoate (**1b**) gave the desired trifluoromethyl ketone **2b** in only lower yield (37%). Functionalities on the benzene ring at the *para*-position were well-tolerated in the KHMDS/glyme system. Halogen groups, such as chloro (**1c**), bromo (**1d**), and reactive iodo (**1e**) substitutions were also tolerated, resulting in the corresponding trifluoromethyl aryl ketones in moderate yields (56–63%) under basic conditions. The alkyl groups of *tert*-butyl (**1f**)- and cyclohexyl (**1g**)-substituted methyl benzoate derivatives, biphenyl benzoate (**1h**), and electron-donating 4-methoxybenzoate, were nicely transformed into aryl trifluoromethyl ketones in moderate to high yields (45–92%). Aryl substrates with a halogen attached at the *meta-* and *ortho-*positions were also accepted to furnish the desired products (**2j**–**m**) in good yields (66–82%). Moreover, di-substituted benzoate (**1n**), sterically demanding methyl adamantly carboxylate (**1o**), and conjugated methyl ester (**1p**) transformed effectively into trifluoromethyl ketones (**2o**–**p**) in moderate yields (50–62%). A gram-scale reaction was also carried out for **1h**, **1n**, and **1p** to furnish **2h**, **2p**, and **2n** in similar isolated yields, 43%, 40%, and 36%, respectively. The double CF_3_ addition product **3** was not observed due to the preferential formation of stable tetrahedral species **I** instead of the CF_3_ ketones **2** in the reaction mixture. However, all the yields were moderate to good. This fact could be explained by the appearance of hydrate products **4** in the ^19^F NMR spectrum of the crude reaction mixture [72], while the hydrates **4** disappeared completely after purification by silica gel column chromatography [73].

Given the relevance of this trifluoromethylation reaction system for drug discovery, we conducted a robustness screening experiment to gain further information on its tolerance to various pharmacophores (Table 2). A range of common nitrogen-containing compounds such as pyridine, pyrazine, 1*H*-pyrazole, 1*H*-indole, 1-methyl-1*H*-indole, piperidine, and piperazine were subjected to screening. Pyridine and piperidine slightly hamper the reaction of **1g** (Table 2, entries 2 and 7, 80–82%). Other nitrogen-containing compounds have more effect on the yield of the reaction of **1g** (Table 2, entries 3–6, 58–72%). Next, a range of common oxygen and sulfur-containing compounds such as furan, tetrahydrofuran, 1,4-dioxane, thiophene, benzo[*b*]thiophene, dibenzo[*b,d*]thiophene, and diphenylsulfane were also screened. These substances also have some effect on the reaction (Table 2, entries 9–15, 63–87%). Besides, silicon-containing compound, trimethyl(phenyl)silane that is more sensitive to fluorine was screened, 79% yield were obtained in this test. To consider the frequency of these motifs in modern pharmaceutical drugs, these tests are necessary, and the resistance of the reaction was also verified from various pharmacophores to be acceptable.

**Table 2:** Tolerance of various pharmacophores under the trifluoromethylation conditions.

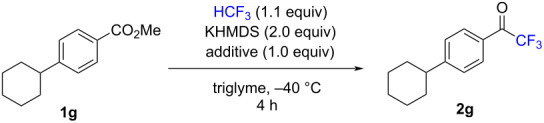								

Entry	Additive	Yield^a^ (%)	Entry	Additive	Yield^a^ (%)	Entry	Additive	Yield^a^ (%)

1	–	92	7	piperidine	80	13	benzo[*b*]thiophene	78
2	pyridine	82	8	piperazine	64	14	dibenzo[*b*,*d*]thiophene	64
3	pyrazine	72	9	furan	87	15	PhSPh	75
4	pyrazole	66	10	THF	64	16	Ph-SiMe_3_	79
5	indole	58	11	1,4-dioxane	63			
6	1-methyl-1*H*-indole	67	12	thiophene	75			

^a^Determined by ^19^F NMR using the crude **2g** with trifluorotoluene as an internal standard.

## Conclusion

In conclusion, the trifluoromethylation of methyl carboxylates to trifluoromethyl ketones is accomplished under basic conditions with fluoroform in triglyme at −40 °C. An equivalent amount of fluoroform was sufficient for this transformation. A wide variety of medicinally attractive aryl and alkyl trifluoromethyl ketones are obtained in good yields by a relatively simple procedure, although the protocol is not applicable to enolizable esters. Fluoroform is an economical feedstock, and methyl esters are readily available inexpensive precursors. Besides, glymes are versatile solvents for chemical processes in industry [74] would the protocol be useful for the industrial extension, although there are still many points to be overcome such as requirements of low temperature, two equivalents of KHMDS. Further application of this “batch protocol” for a “continuous-flow microreactor” reaction is now ongoing in our laboratory towards industrial collaboration.

## Experimental

A test tube containing **1** (0.4 mmol) in triglyme (0.7 mL) was charged with HCF_3_ (9.9 mL, 1.1 equiv, measured by a syringe, see the picture in Supporting Information File 1, Figure S1) by cooling in liquid nitrogen under vacuum. KHMDS (160 mg, 2.0 equiv) in triglyme (commercial grade, without drying, 0.3 mL) was added at −40 °C under nitrogen atmosphere, and the reaction mixture was stirred at the same temperature for 4 h. Thereafter, 1 M HCl aq (1.0 mL) was added, and the aqueous layer was extracted with CH_2_Cl_2_ (1.0 mL × 3). The combined organic layer was washed with brine, dried over Na_2_SO_4_, concentrated under reduced pressure, and purified by column chromatography on silica gel to give products **2**.

## Supporting Information

File 1 Optimization of reaction conditions, general procedure and product characterization data.
