# Supplementary material for: Synthesis of trifluoromethyl ketones by nucleophilic trifluoromethylation of esters under a fluoroform/KHMDS/triglyme system
Source: Beilstein J Org Chem. 2021 Feb 12;17:431–8. doi: 10.3762/bjoc.17.39 (PMC7884878; doi:10.3762/bjoc.17.39)

BEILSTEIN JOURNAL OF ORGANIC CHEMISTRY

Supporting Information

for

Synthesis of trifluoromethyl ketones by nucleophilic trifluoromethylation of esters under a fluoroform/KHMDS/triglyme system

Yamato Fujihira, Yumeng Liang, Makoto Ono, Kazuki Hirano, Takumi Kagawa and Norio Shibata

Beilstein J. Org. Chem. **2021,** *17,* 431–438. doi:10.3762/bjoc.17.39

Optimization of reaction conditions, general procedure and product characterization data

License and Terms: This is a supporting information file under the terms of the Creative Commons Attribution License (https://creativecommons.org/licenses/by/4.0). Please note that the reuse, redistribution and reproduction in particular requires that the author(s) and source are credited and that individual graphics may be subject to special legal provisions.
The license is subject to the *Beilstein Journal of Organic Chemistry* terms and conditions: (https://www.beilstein-journals.org/bjoc/terms)

Contents

1. General information. S3

2. Additional experiments for the reaction conditions S4

3. Experiments for the reaction time S5

4. General procedure and product characterization data for 2. ..... S6

5. Supplementary references S13

6. ^{1}H NMR spectra, ^{19}F NMR spectra and ^{13}C NMR spectra S14

1. General information.

All reactions were performed in oven-dried glassware. Solvents were transferred via syringe. All reactions were monitored by thin-layer chromatography (TLC) carried out on 0.25 mm Merck silica-gel (60-F254). Column chromatography was carried out in a column packed with silica-gel 60N spherical neutral size 40–50 μm. The ^{1}H NMR (300 MHz), ^{19}F NMR (282 MHz), and ^{13}C NMR (125.8 MHz) spectra in a $CDCl_3$ solution were recorded on a Varian Mercury 300 or a Bruker Avance 500 instrument. Chemical shifts (δ) are expressed in ppm downfield from internal TMS or hexafluorobenzene. GC–MS was recorded on JEOL JMS-Q1050GC/Agilent7890. High resolution mass spectrometry (HRMS) was recorded on a SHIMADZU GCMS-QP5050A (EIMS) spectrometer. Infrared spectra were recorded on a JASCO FT/IR-4100 spectrometer. Melting points were recorded on a BÜCHI M-565 apparatus. Commercially available chemicals were obtained from Ark Pharm Inc., Aldrich Chemical Co., Nacalai tesque, TCI, or Wako, and were used as received unless otherwise stated.

2. Additional experiments for the reaction conditions

Table S1. Additional experiments for the reaction conditions with **1a**

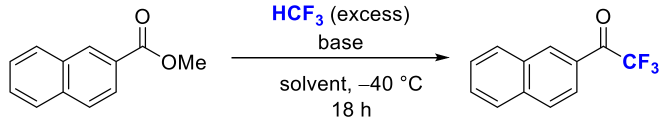

entry	base (equiv)	solvent	temp. (°C)	yield[a] (%)
1	*t*-BuOK (2.0)	THF	-40	5
2	*t*-BuOK (2.0)	toluene	-40	0
3	*t*-BuOK (2.0)	triglyme	-40	29
4	*t*-BuOK (2.0)	triglyme	-60	22
5	*t*-BuOK (3.0)	triglyme	-40	16
6	*t*-BuOK (4.0)	triglyme	-40	25
7	*t*-BuOK (4.0)	triglyme	-60	6
8	KHMDS (2.0)	triglyme	-40	59
9	KHMDS (2.0)	triglyme	-60	54
10	KHMDS (1.2)	triglyme	-60	37
11	PhOK (2.0)	triglyme	-40	0
12	*t*-BuOK (2.0)	diglyme	-40	5
13	KHMDS (2.0)	diglyme	-40	29
14	KHMDS (2.0)	diglyme	-60	23
15	*t*-BuOK (2.0)	tetraglyme	-40	15
16	KHMDS (2.0)	tetraglyme	-40	57
17[b]	KHMDS (2.0)	triglyme	-40	64
18[b, c]	KHMDS (2.0)	triglyme	-40	75 (71)[d]

[a] Determined by ^{19}F NMR yield using the crude mixture with trifluorotoluene as the internal standard.

[b] The reaction time was 4 h. [c] HCF_3 was 1.1 equiv. [d] Isolated yield

3. Experiments for the reaction time

Table S2. Experiments for the reaction time with **1c** and **1p**

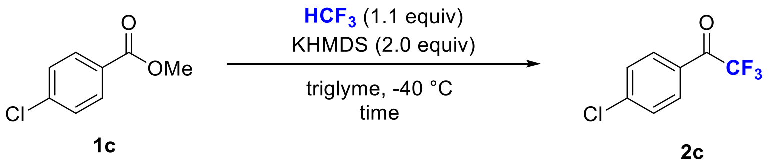

time (h)	1	2	**4**	6	8	10	12	18
^{19}F NMR yield (%)	19	29	**42**	38	28	26	19	15

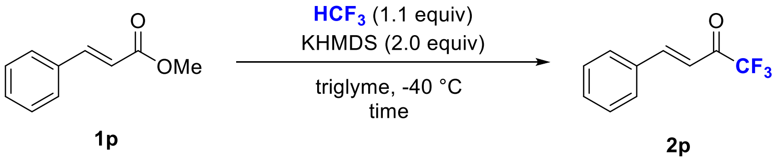

time (h)	1	2	**4**	6	8	10	12	18
^{19}F NMR yield (%)	26	33	**49**	30	32	33	25	23

4. General procedure and product characterization data for 2.

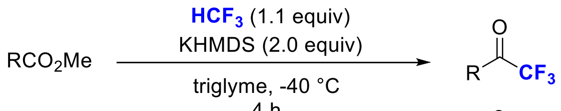

1 **2**

Scheme S1. General procedure for trifluoromethyl compounds **2** from methyl ester **1.**

General procedure:

A test tube containing **1** (0.4 mmol) in triglyme (0.7 mL) was charged with HCF_3 (9.9 mL, 1.1 equiv) by a syringe (**Figures S1**) by cooling in liquid nitrogen under vacuum. KHMDS (160 mg, 2.0 equiv) in triglyme (0.3 mL) was added at –40 °C, and the reaction mixture was stirred at the same temperature for 4 h. Thereafter, 1M HCl aq. (1.0 mL) was added, and the aqueous layer was extracted with CH_2Cl_2 (1.0 mL × 3). The combined organic layer was washed with brine, dried over Na_2SO_4, concentrated under reduced pressure, and purified by column chromatography on silica gel to give products **2**. The commercial grade of triglyme was used without further drying.

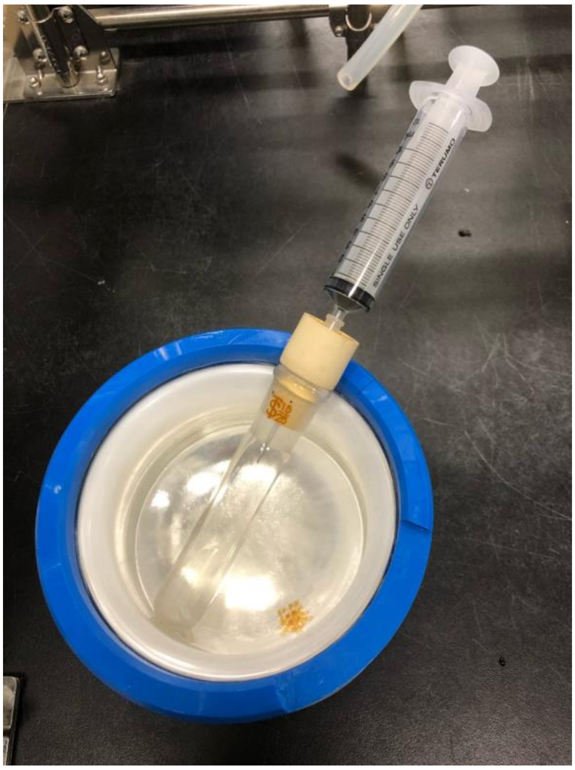

Figure S1. Addition of HCF_3 by a syringe

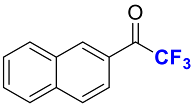

2,2,2-Trifluoro-1-(naphthalen-2-yl)ethan-1-one (2a).

Following the general procedure, the mixture was purified by column chromatography on silica gel (^{n}hexane/diethyl ether = 5/1) to give **2a** (63.7 mg, 71% yield) as a yellow solid.

^{1}H NMR (300 MHz, Chloroform-*d*) δ: 8.61 (s, 1H), 8.07-7.88 (m, 4H), 7.71-7.58 (m, 2H) ppm; **^{19}F NMR** (282 MHz, Chloroform-*d*) δ: −71.22 (s, 3F) ppm; **GC-MS** (EI, *m/z*): 127 $[M\text{-}COCF_3]^+$ (100%), 155 $[M\text{-}CF_3]^+$ (79%), 224 $[M]^+$ (17%).

Spectroscopic data was agreement with the literature[1].

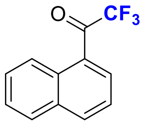

2,2,2-Trifluoro-1-(naphthalen-1-yl)ethan-1-one (2b)

Following the general procedure, the mixture was purified by column chromatography on silica gel (^{n}hexane only) to give **2b** (30.3 mg, 34% yield) as a colorless oil.

^{1}H NMR (300 MHz, Chloroform-*d*) δ: 8.84 (d, *J* = 8.5 Hz, 1H), 8.22-8.16 (m, 2H), 7.95 (d, *J* = 7.9 Hz, 1H), 7.74-7.57 (m, 3H) ppm; **^{19}F NMR** (282 MHz, Chloroform-*d*) δ: −70.63 (s, 3F) ppm; **GC-MS** (EI, *m/z*): 127 $[M\text{-}COCF_3]^+$ (100%), 155 $[M\text{-}CF_3]^+$ (79%), 224 $[M]^+$ (21%).

Spectroscopic data was agreement with the literature[1].

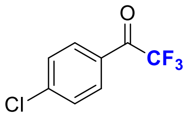

1-(4-Chlorophenyl)-2,2,2-trifluoroethan-1-one (2c)

Following the general procedure, the mixture was purified by column chromatography on silica gel (^{n}hexane/DCM = 1/1) to give **2c** (43.2 mg, 52% yield) as a colorless oil.

^{1}H NMR (300 MHz, Chloroform-*d*) δ: 8.01 (d, *J* = 8.2 Hz, 2H), 7.53 (d, *J* = 8.5 Hz, 2H) ppm; **^{19}F NMR** (282 MHz, Chloroform-*d*) δ: −71.98 (s, 3F) ppm; **GC-MS** (EI, *m/z*): 111 $[M\text{-}COCF_3]^+$ (^{35}Cl, 57%), 113 $[M\text{-}COCF_3]^+$ (^{37}Cl, 15%), 139 $[M\text{-}CF_3]^+$ (^{35}Cl, 100%), 141 $[M\text{-}CF_3]^+$ (^{37}Cl, 31%), 208 $[M]^+$ (^{35}Cl, 6%), 210 $[M]^+$ (^{37}Cl, 1%).

Spectroscopic data was agreement with the literature[1].

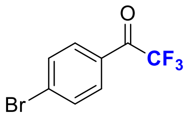

1-(4-Bromophenyl)-2,2,2-trifluoroethan-1-one (2d)

Following the general procedure, the mixture was purified by column chromatography on silica gel (^{n}hexane/DCM = 1/1) to give **2d** (39.5 mg, 39% yield) as a colorless oil.

^{1}H NMR (300 MHz, Chloroform-*d*) δ: 7.94 (d, *J* = 7.9 Hz, 2H), 7.71 (d, *J* = 8.8 Hz, 2H) ppm; **^{19}F NMR** (282 MHz,

Chloroform-*d*) δ: −72.04 (s, 3F) ppm; **GC-MS** (EI, *m/z*): 155 $[M{-}COCF_3]^+$ (^{79}Br, 49%), 157 $[M{-}COCF_3]^+$ (^{81}Br, 49%), 183 $[M{-}CF_3]^+$ (^{79}Br, 100%), 185 $[M{-}CF_3]^+$ (^{81}Br, 96%), 252 $[M]^+$ (^{79}Br, 13%), 254 $[M]^+$ (^{81}Br, 12%).

Spectroscopic data was agreement with the literature[1].

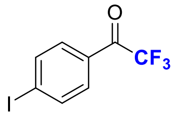

2,2,2-Trifluoro-1-(4-iodophenyl)ethan-1-one (2e)

Followed the general procedure, purified by column chromatography on silica gel (^{n}hexane/acetone = 4/1) to give **2e** (61.2 mg, 51% yield) as a yellow solid.

^{1}H NMR (300 MHz, Chloroform-*d*) δ: 7.94 (d, *J* = 7.9 Hz, 2H), 7.76 (d, *J* = 7.9 Hz, 2H) ppm; **^{19}F NMR** (282 MHz, Chloroform-*d*) δ: −72.04 (s, 3F) ppm; **GC-MS** (EI, *m/z*): 203 $[M\text{-}COCF_3]^+$ (35%), 231 $[M\text{-}CF_3]^+$ (100%), 300 $[M]^+$ (14%).

Spectroscopic data was agreement with the literature[6].

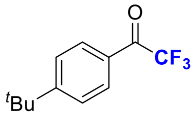

1-(4-(*tert*-Butyl)phenyl)-2,2,2-trifluoroethan-1-one (2f)

Following the general procedure, the mixture was purified by column chromatography on silica gel (^{n}hexane/DCM = 4/1) to give **2f** (35.2 mg, 38% yield) as a white solid.

^{1}H NMR (300 MHz, Chloroform-*d*) δ: 8.02 (d, *J* = 8.2 Hz, 2H), 7.56 (d, *J* = 8.5 Hz, 2H), 1.36 (s, 9H) ppm; **^{19}F NMR** (282 MHz, Chloroform-*d*) δ: −71.83 (s, 3F) ppm; **HRMS** (EI, m/z) calcd. for $C_{12}H_{13}OF_3$ $[M]^+$: 230.0918 Found: 230.0916.

Spectroscopic data was agreement with the literature[3].

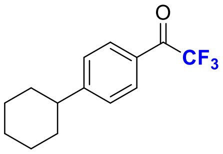

1-(4-Cyclohexylphenyl)-2,2,2-trifluoroethan-1-one (2g)

Following the general procedure, the mixture was purified by column chromatography on silica gel (^{n}hexane/DCM = 9/1) to give **2g** (78.5 mg, 77% yield) as a colorless oil.

^{1}H NMR (300 MHz, Chloroform-*d*) δ: 8.01 (d, *J* = 7.9 Hz, 2H), 7.38 (d, *J* = 8.2 Hz, 2H), 2.61 (s, 1H), 1.89-1.76 (m, 5H), 1.51-1.26 (m, 5H) ppm; **^{19}F NMR** (282 MHz, Chloroform-*d*) δ: −71.76 (s, 3F) ppm; **GC-MS** (EI, *m/z*): 187 $[M\text{-}CF_3]^+$ (100%).

Spectroscopic data was agreement with the literature[4].

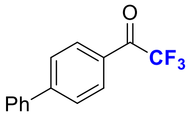

1-([1,1'-Biphenyl]-4-yl)-2,2,2-trifluoroethan-1-one (2h)

Following the general procedure, the mixture was purified by column chromatography on silica gel (^{n}hexane/diethyl ether = 9/1) to give **2h** (38.1 mg, 38% yield) as a white solid.

Gram-scale synthesis: Followed by the general procedure, used **1h** (1g, 4.71 mmol), HCF_3 (excess), KHMDS (1.88 g, 9.42 mmol, 2.0 equiv) and triglyme (24 mL) to give **2h** (506 mg, 43%) as a white solid.

^{1}H NMR (300 MHz, Chloroform-*d*) δ: 8.16 (d, *J* = 7.9 Hz, 2H), 7.79-7.43 (m, 7H) ppm; **^{19}F NMR** (282 MHz, Chloroform-*d*) δ: −71.82 (s, 3F) ppm; **GC-MS** (EI, *m*/*z*): 152 $[M\text{-}COCF_3]^+$ (79%), 181 $[M\text{-}CF_3]^+$ (100%), 250 $[M]^+$ (30%).

Spectroscopic data was agreement with the literature[1]

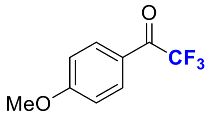

2,2,2-Trifluoro-1-(4-methoxyphenyl)ethan-1-one (2i)

Following the general procedure, the mixture was purified by column chromatography on silica gel (^{n}hexane/diethyl ether = 9/1) to give **2i** (36.9 mg, 45% yield) as a colorless oil.

^{1}H NMR (300 MHz, Chloroform-*d*) δ: 8.06 (d, *J* = 8.5 Hz, 2H), 7.01 (d, *J* = 9.1 Hz, 2H), 3.92 (s, 3H) ppm; **^{19}F NMR** (282 MHz, Chloroform-*d*) δ: −71.47 (s, 3F) ppm; **GC-MS** (EI, *m*/*z*): 135 $[M\text{-}CF_3]^+$ (82%), 152 $[M\text{-}CF_3\text{+}OH]^+$ (100%).

Spectroscopic data was agreement with the literature[1].

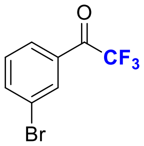

1-(3-Bromophenyl)-2,2,2-trifluoroethan-1-one (2j)

Following the general procedure, the mixture was purified by column chromatography on silica gel (^{n}hexane/DCM = 2/1) to give **2j** (60.5 mg, 60% yield) as a colorless oil.

^{1}H NMR (300 MHz, Chloroform-d) δ: 8.20 (s, 1H), 8.01 (d, *J* = 7.3 Hz, 1H), 7.85 (d, *J* = 8.8 Hz, 1H), 7.45 (t, *J* = 7.9 Hz, 1H) ppm; **^{19}F NMR** (282 MHz, Chloroform-d) δ: −72.05 (s, 3F) ppm; **GC-MS** (EI, *m*/*z*): 155 $[M-COCF_3]^+$ (^{79}Br, 68%), 157 $[M-COCF_3]^+$ (^{81}Br, 63%), 183 $[M-CF_3]^+$ (^{79}Br, 100%), 185 $[M-CF_3]^+$ (^{81}Br, 97%), 252 $[M]^+$ (^{79}Br, 14%),

254 [M]$^+$ (^{81}Br, 15%).

Spectroscopic data was agreement with the literature[9].

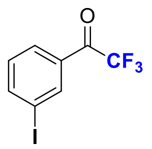

2,2,2-Trifluoro-1-(3-iodophenyl)ethan-1-one (2k)

Following the general procedure, the mixture was purified by column chromatography on silica gel (^{n}hexane/ethyl acetate = 4/3) to give **2k** (90.3 mg, 75% yield) as a yellow oil.

^{1}H NMR (300 MHz, Chloroform-*d*) δ: 8.39 (s, 1H), 8.06-8.03 (m, 2H), 7.34-7.26 (m, 1H) ppm; **^{19}F NMR** (282 MHz, Chloroform-*d*) δ: −72.01 (s, 3F) ppm; **GC-MS** (EI, *m*/*z*): 203 [M-COCF$_3$]$^+$ (49%), 231 [M-CF$_3$]$^+$ (100%), 300 [M]$^+$ (31%).

Spectroscopic data was agreement with the literature[7].

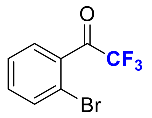

1-(2-Bromophenyl)-2,2,2-trifluoroethan-1-one (2l)

Followed the general procedure, purified by column chromatography on silica gel (^{n}hexane/ethyl acetate = 5/1) to give **2l** (56.7 mg, 56% yield) as a colorless oil.

^{1}H NMR (300 MHz, Chloroform-*d*) δ: 7.77-7.70 (m, 2H), 7.48-7.46 (m, 2H) ppm; **^{19}F NMR** (282 MHz, Chloroform-*d*) δ: −73.50 (s, 3F) ppm; **GC-MS** (EI, *m*/*z*): 155 [M−COCF$_3$]$^+$ (^{79}Br, 51%), 157 [M−COCF$_3$]$^+$ (^{81}Br, 48%), 183 [M−CF$_3$]$^+$ (^{79}Br, 100%), 185 [M−CF$_3$]$^+$ (^{81}Br, 98%), 252 [M]$^+$ (^{79}Br, 5%), 254 [M]$^+$ (^{81}Br, 3%).

Spectroscopic data was agreement with the literature[2].

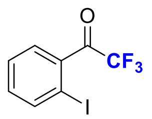

2,2,2-Trifluoro-1-(2-iodophenyl)ethan-1-one (2m)

Following the general procedure, the mixture was purified by column chromatography on silica gel (^{n}hexane/DCM = 3/1) to give **2m** (92.6 mg, 77% yield) as a yellow solid.

^{1}H NMR (300 MHz, Chloroform-*d*) δ: 8.09 (d, *J* = 7.6 Hz, 1H), 7.74 (d, *J* = 6.7 Hz, 1H), 7.51 (t, *J* = 7.6 Hz, 1H), 7.29 (t, *J* = 7.6 Hz, 1H) ppm; **^{19}F NMR** (282 MHz, Chloroform-*d*) δ: −72.77 (s, 3F) ppm; **^{13}C NMR** (126 MHz, Chloroform-*d*) δ: 182.4 (q, *J* = 35.7 Hz), 142.4, 134.3, 130.2, 130.1, 128.1, 115.5 (q, *J* = 292.1 Hz), 94.0 ppm; **ATR-FTIR** (KBr): ν = 3431, 3092, 1469, 1432, 1323, 1279, 1052, 1020, 792, 755, 698, 676, 637, 460, 422 cm^{-1};

mp: 40.2-40.7 °C; **HRMS** (EI, m/z) calcd. for $C_8H_4OF_3I$ [M]$^+$: 299.9259 Found: 299.9261.

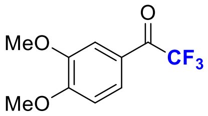

1-(3,4-Dimethoxyphenyl)-2,2,2-trifluoroethan-1-one (2n)

Following the general procedure, the mixture was purified by column chromatography on silica gel (^{n}hexane/diethyl ether = 9/1) to give **2n** (39.0 mg, 42% yield) as a white solid.

Gram-scale synthesis: Followed by the general procedure, used **1n** (1g, 5.10 mmol), HCF_3 (excess), KHMDS (2.03 g, 10.2 mmol, 2.0 equiv) and triglyme (26 mL) to give **2n** (478 mg, 40%) as a white solid.

^{1}H NMR (300 MHz, Chloroform-*d*) δ: 7.74 (d, *J* = 8.2 Hz, 1H), 7.58 (s, 1H), 6.96 (d, *J* = 8.5 Hz, 1H), 4.00 (s, 3H), 3.96 (s, 3H) ppm; **^{19}F NMR** (282 MHz, Chloroform-*d*) δ: −70.90 (s, 3F) ppm; **GC-MS** (EI, *m*/*z*): 165 [M-CF_3]$^+$ (100%), 234 [M]$^+$ (51%).

Spectroscopic data was agreement with the literature[5].

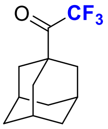

1-((3*r*,5*r*,7*r*)-Adamantan-1-yl)-2,2,2-trifluoroethan-1-one (2o)

Followed the general procedure, purified by column chromatography on silica gel (^{n}hexane/ethyl acetate = 9/1) to give **2o** (54.0 mg, 58% yield) as a colorless oil.

^{1}H NMR (300 MHz, Chloroform-*d*) δ: 3.65 (s, 2H), 2.09-1.67 (m, 13H) ppm; **^{19}F NMR** (282 MHz, Chloroform-d) δ: −72.66 (s, 3F) ppm; **GC-MS** (EI, *m*/*z*): 135 [M−$COCF_3$]$^+$ (100%).

Spectroscopic data was agreement with the literature[8].

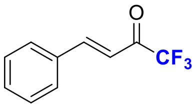

(*E*)-1,1,1-Trifluoro-4-phenylbut-3-en-2-one (2p)

Following the general procedure, the mixture was purified by column chromatography on silica gel (^{n}hexane/DCM = 10/1) to give **2p** (32.8 mg, 41% yield) as a yellow oil.

Gram-scale synthesis: Followed by the general procedure, used **1p** (1g, 6.17 mmol), HCF_3 (excess), KHMDS (2.45 g, 12.3 mmol, 2.0 equiv) and triglyme (31 mL) to give **2p** (445 mg, 36% yield) as a yellow oil.

^{1}H NMR (300 MHz, Chloroform-*d*) δ: 7.97 (d, *J* = 15.8 Hz, 1H), 7.64 (d, *J* = 6.7 Hz, 2H), 7.50-7.43 (m, 3H), 7.02 (d, *J* = 15.8 Hz, 1H); **^{19}F NMR** (282 MHz, Chloroform-*d*) δ: −78.05 (s, 3F) ppm; **GC-MS** (EI, *m*/*z*): 103 [M-$COCF_3$]$^+$ (79%), 131 [M-CF_3]$^+$ (100%), 200 [M]$^+$ (39%).

Spectroscopic data was agreement with the literature[5].

5. Supplementary references

1. Xing Yi, Ya-Fang Cao, Xing Wang, Hui Xu, Shu-Rong Ban, Hui-Xiong Dai. Palladium-catalyzed fluoroacylation of (Hetero)arylboronic acid with fluorothioacetates at ambient temperature. *Tetrahedron Lett.*, **2020**, *61*, 151780.
2. James P. Phelan, Rebecca J. Wiles, Simon B. Lang, Christopher B. Kelly, and Gary A. Molander. Rapid Access to Diverse, Trifluoromethyl-Substituted Alkenes Using Complementary Strategies., *Chem. Sci.*, **2018**, *9*, 3215–3220.
3. Fengxiang Zhu, Guangfu Yang, Shaolin Zhou, Xiao-Feng Wu. Palladium-catalyzed carbonylative coupling of aryl iodides with an organocopper reagent: a straightforward procedure for the synthesis of aryl trifluoromethyl ketones., *RSC Adv.*, **2016**, *6*, 57070.
4. Xiangyu Guo and Chao-Jun Li. Ruthenium-Catalyzed Para-Selective Oxidative Cross-Coupling of Arenes and Cycloalkanes. *Org. Lett.*, **2011**, *13*, 4977–4979.
5. Paweł Czerwiński, Edyta Molga, Prof. Luigi Cavallo, Dr. Albert Poater, Dr. Michał Michalak. NHC–Copper(I) Halide‐Catalyzed Direct Alkynylation of Trifluoromethyl Ketones on Water. *Chem. Eur. J.*, **2016**, *22*, 8089–8094.
6. Xiao Xu, Qing-Qiang Min, Na Lia and Feng Liu. Visible light-promoted umpolung coupling of aryl tri-/difluoroethanones with 2-alkenylpyridines. *Chem. Commun*., **2018**, *54*, 11017.
7. Christopher B. Kelly, Michael A. Mercadante, Emma R. Carnaghan, Matthew J. Doherty, Diana C. Fager, John J. Hauck, Allyson E. MacInnis, Leon J. Tilley, and Nicholas E. Leadbeater. Synthesis of Perfluoroalkyl-Substituted Vinylcyclopropanes by Way ofEnhanced Neighboring Group Participation. *Eur. J. Org. Chem.* **2015**, 4071–4076.
8. Pei Zhang, Haigen Shen, Lin Zhu, Weiguo Cao, and Chaozhong Li. Radical C(sp2)−H Trifluoromethylation of Aldehydes in Aqueous Solution. *Org. Lett.*, **2018**, *20*, 7062−7065.
9. Zhaomeng Han, Sihan Chen, Yongjun Tu, Xiongdong Lian, and Gongyong Li. Fluoroform: an Efficient Precursor for the Trifluoromethylation of Aromatic Esters by Sodium Diisopropylamide with Trialkylamines. *Eur. J. Org. Chem.* **2019**, 4658–4661.

6. ^{1}H NMR spectra, ^{19}F NMR spectra and ^{13}C NMR spectra

^{1}H NMR (300 MHz, Chloroform-*d*) of **2a**

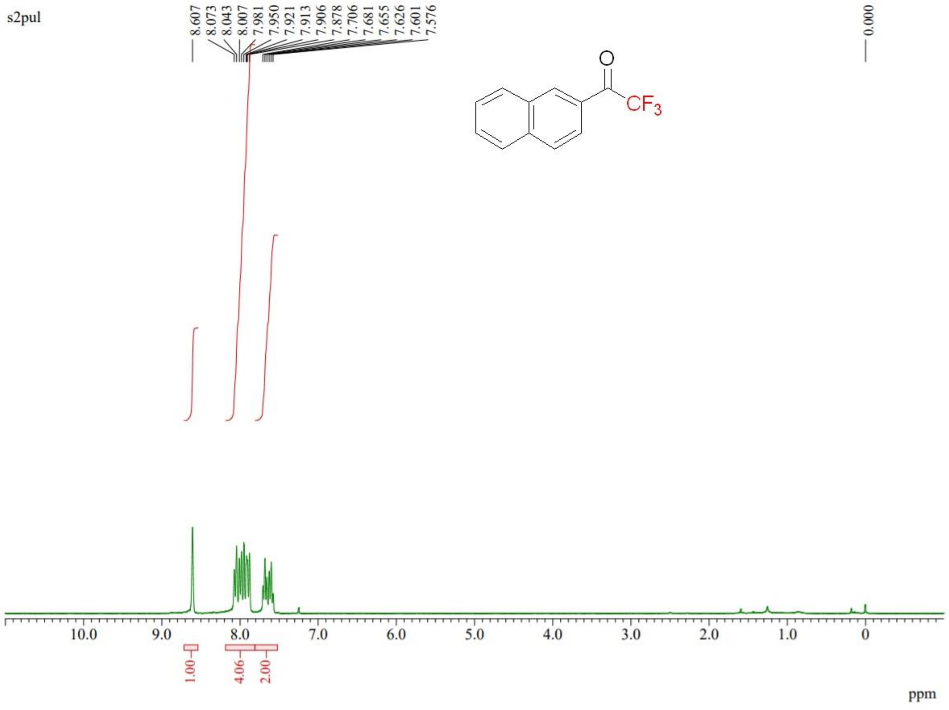

^{19}F NMR (282 MHz, Chloroform-*d*) of **2a**

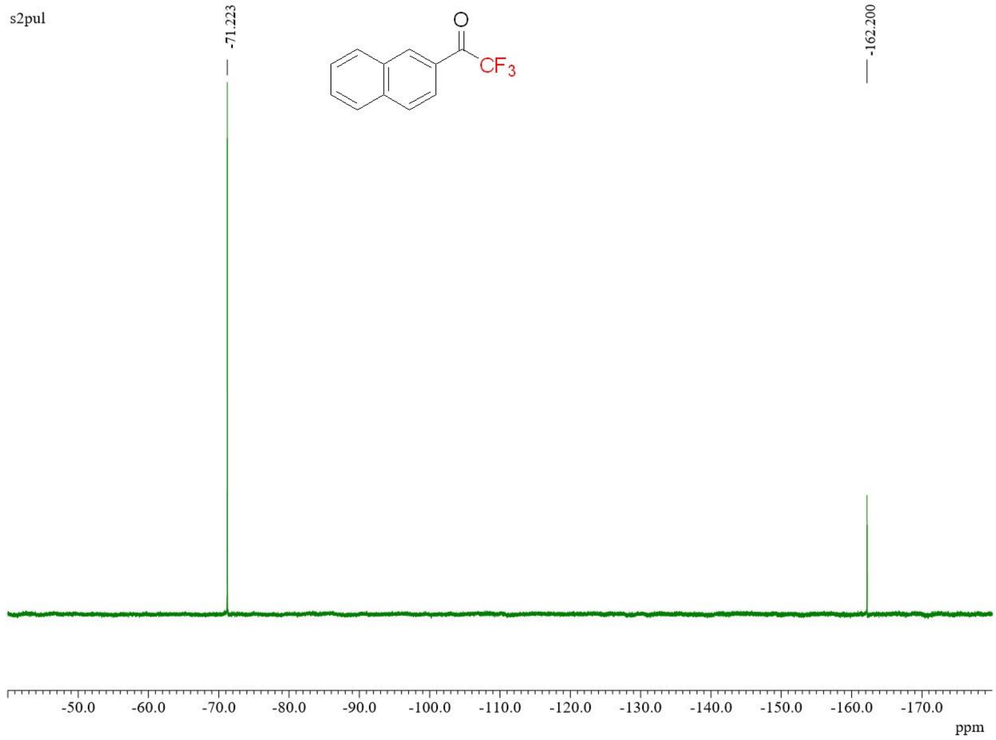

^{1}H NMR (300 MHz, Chloroform-*d*) of **2b**

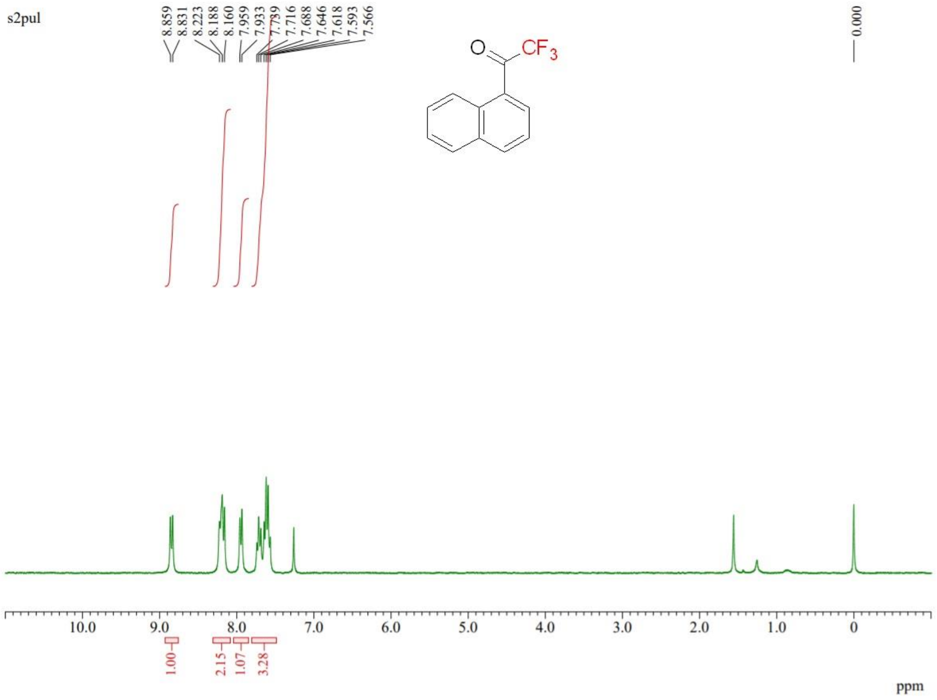

^{19}F NMR (282 MHz, Chloroform-*d*) of **2b**

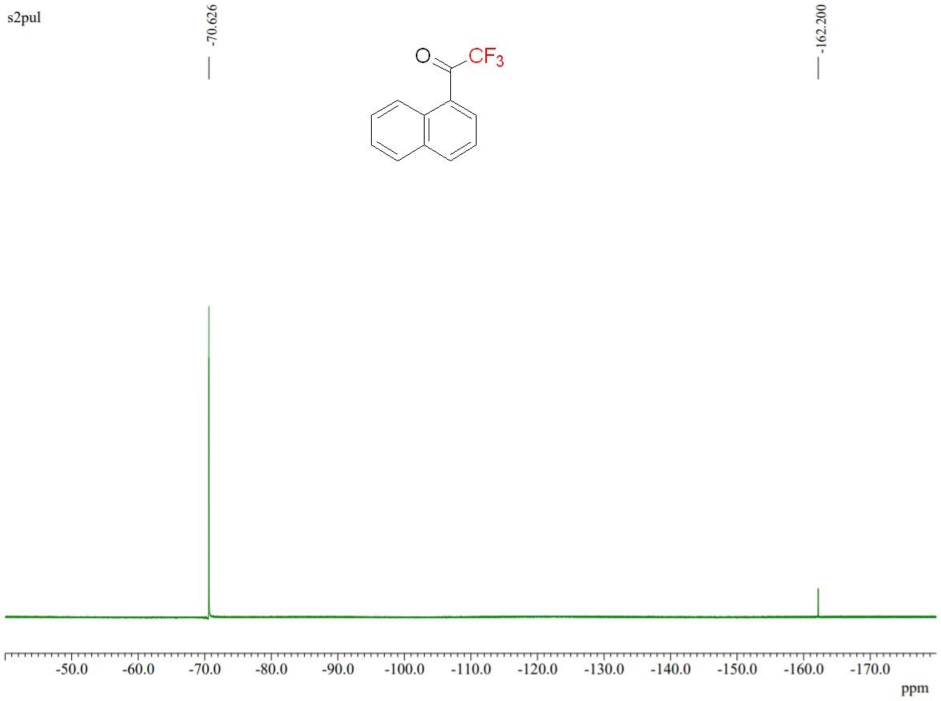

^{1}H NMR (300 MHz, Chloroform-*d*) of **2c**

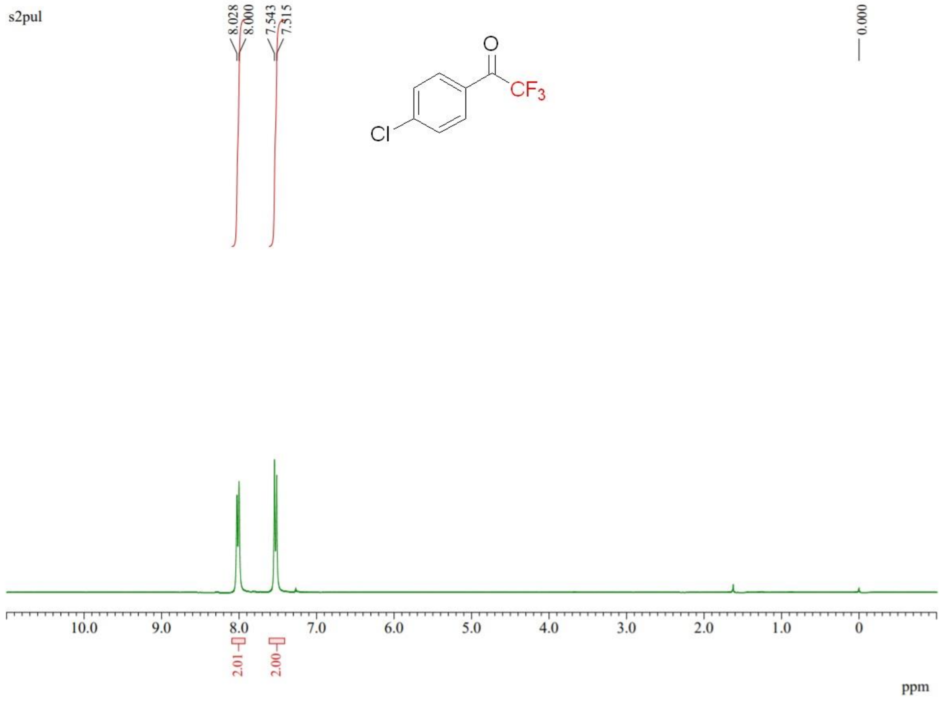

^{19}F NMR (282 MHz, Chloroform-*d*) of **2c**

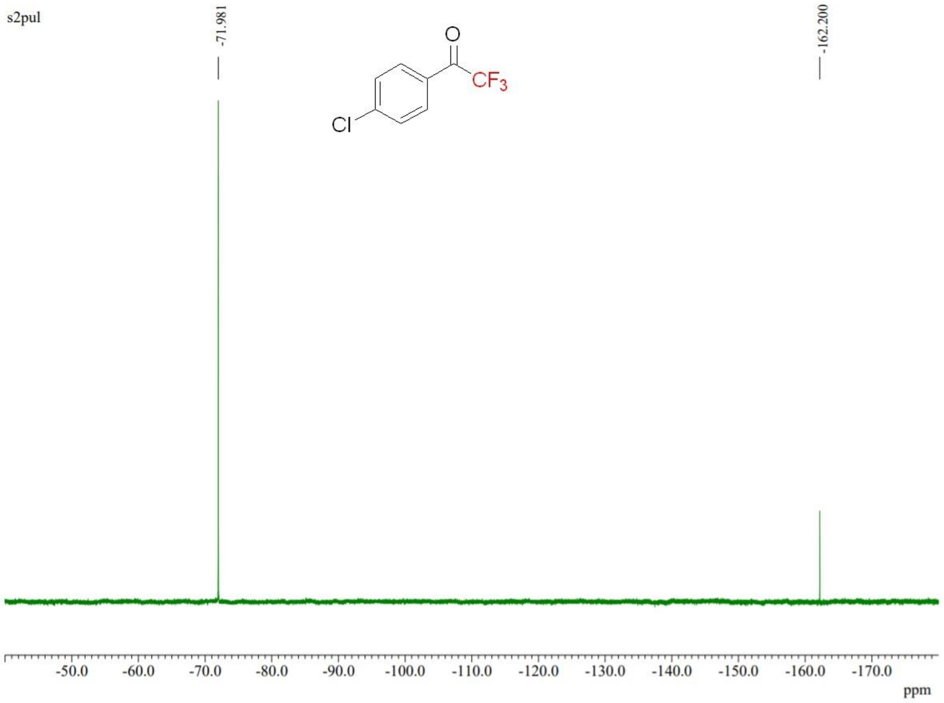

^{1}H NMR (300 MHz, Chloroform-*d*) of **2d**

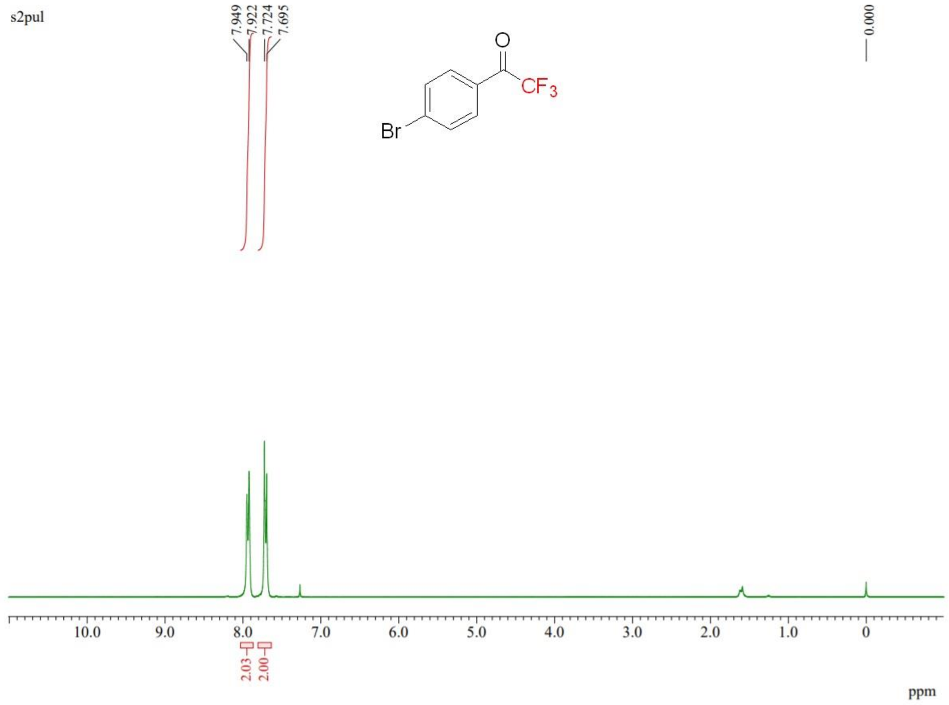

^{19}F NMR (282 MHz, Chloroform-*d*) of **2d**

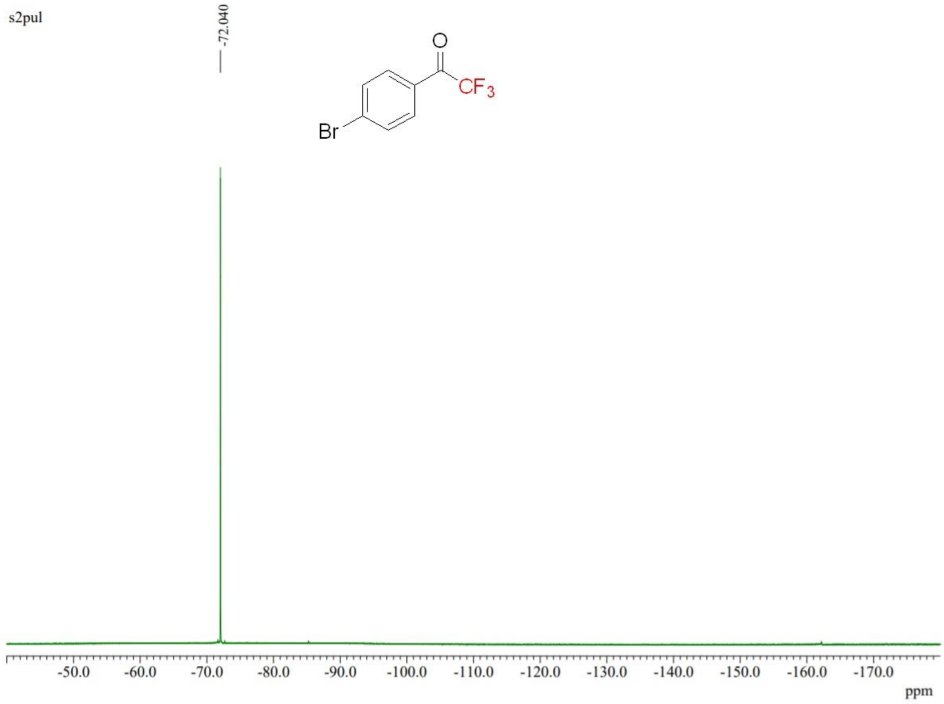

^{1}H NMR (300 MHz, Chloroform-*d*) of **2e**

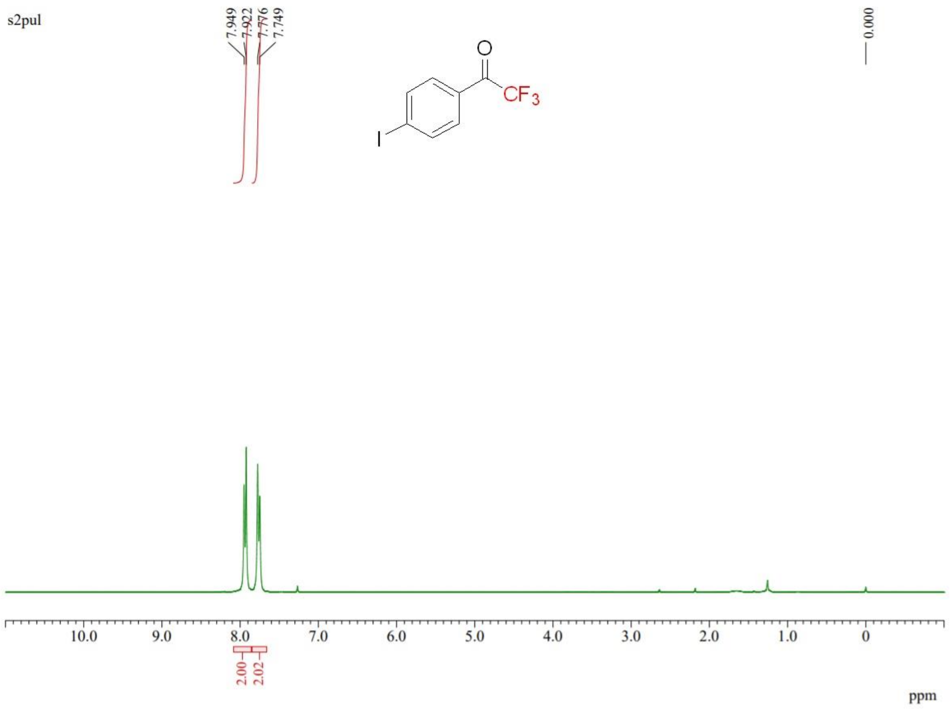

^{19}F NMR (282 MHz, Chloroform-*d*) of **2e**

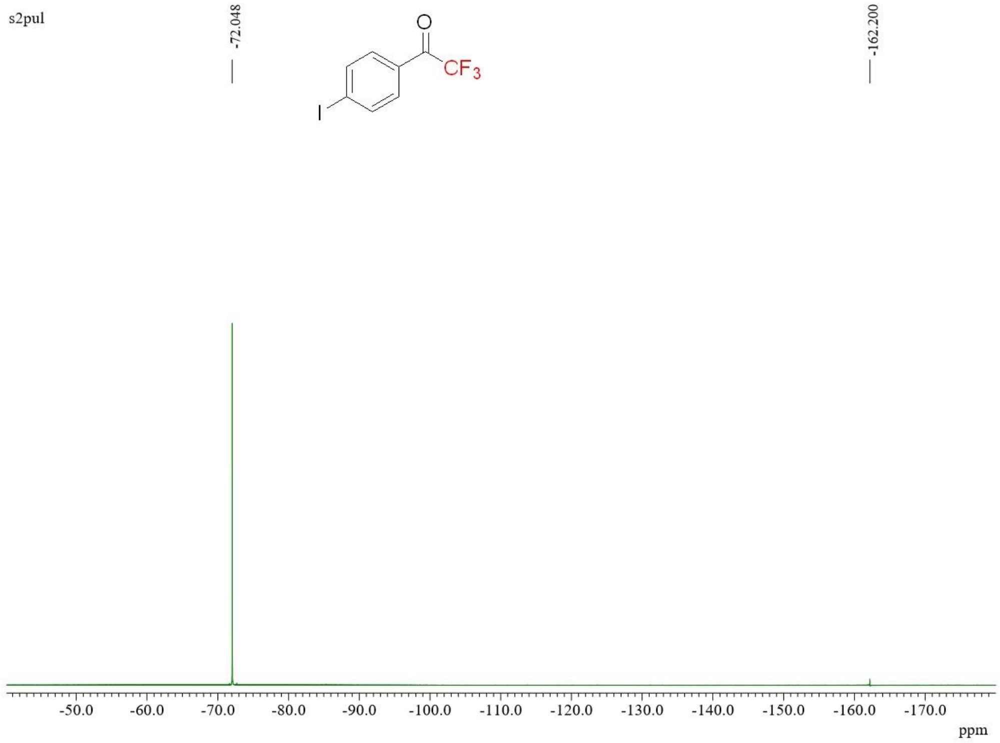

^{1}H NMR (300 MHz, Chloroform-*d*) of **2f**

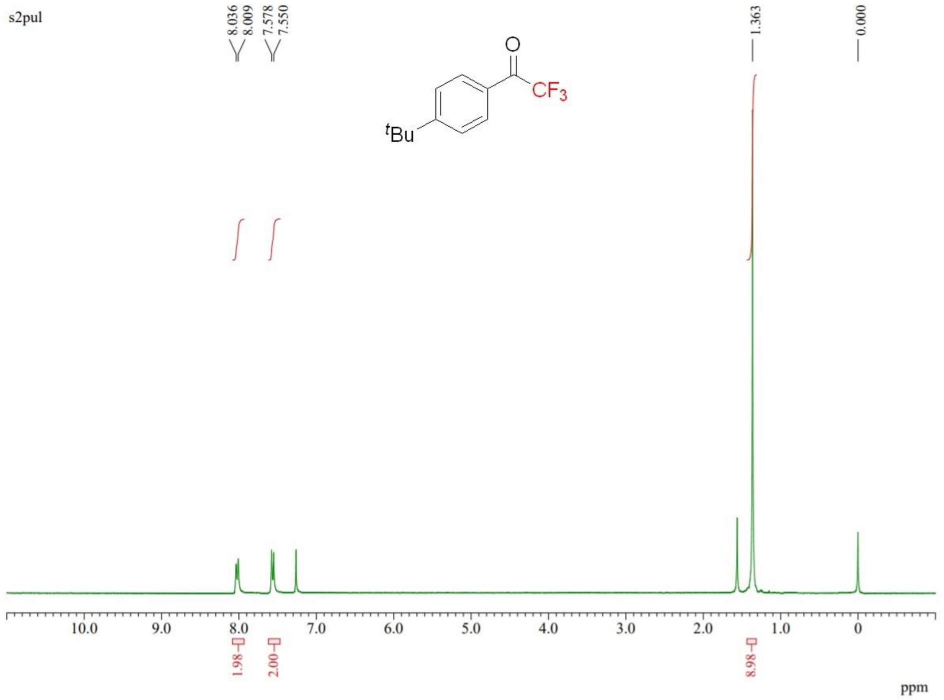

^{19}F NMR (282 MHz, Chloroform-*d*) of **2f**

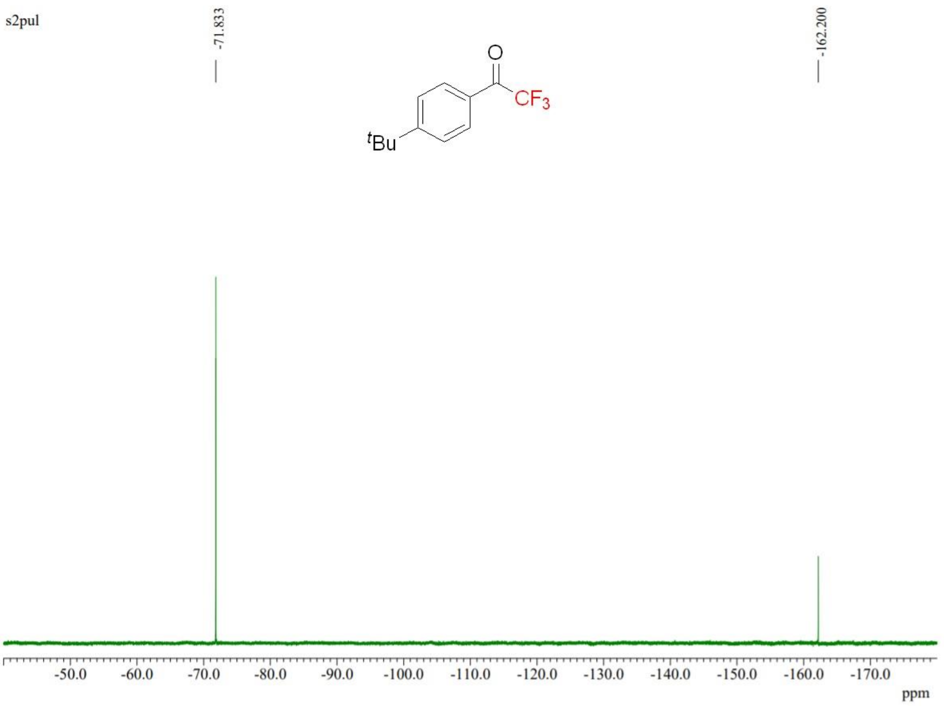

^{1}H NMR (300 MHz, Chloroform-*d*) of **2g**

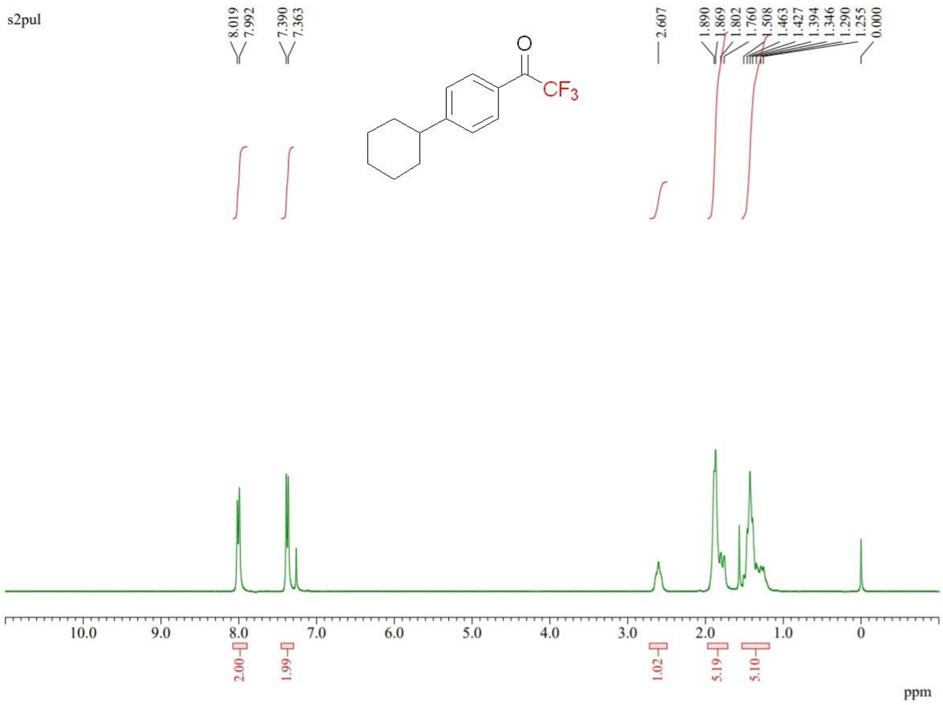

^{19}F NMR (282 MHz, Chloroform-*d*) of **2g**

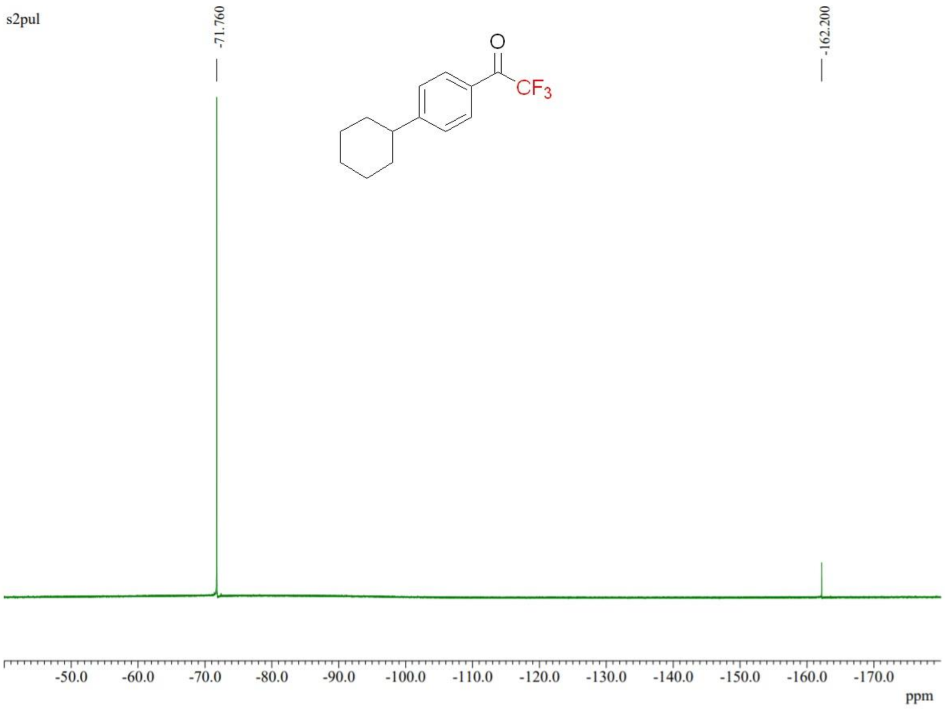

^{1}H NMR (300 MHz, Chloroform-*d*) of **2h**

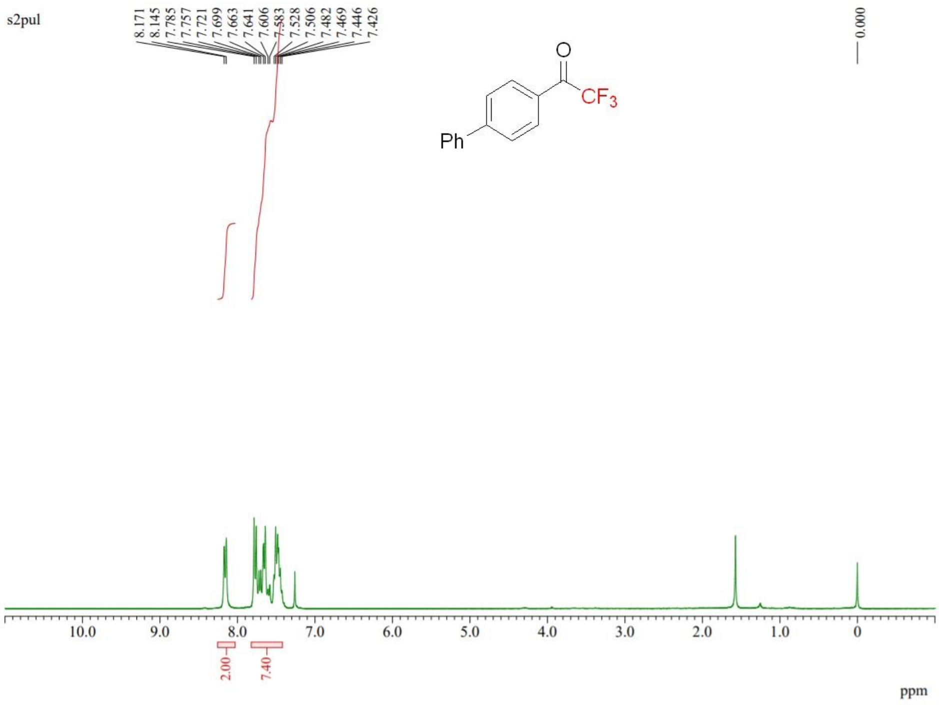

^{19}F NMR (282 MHz, Chloroform-*d*) of **2h**

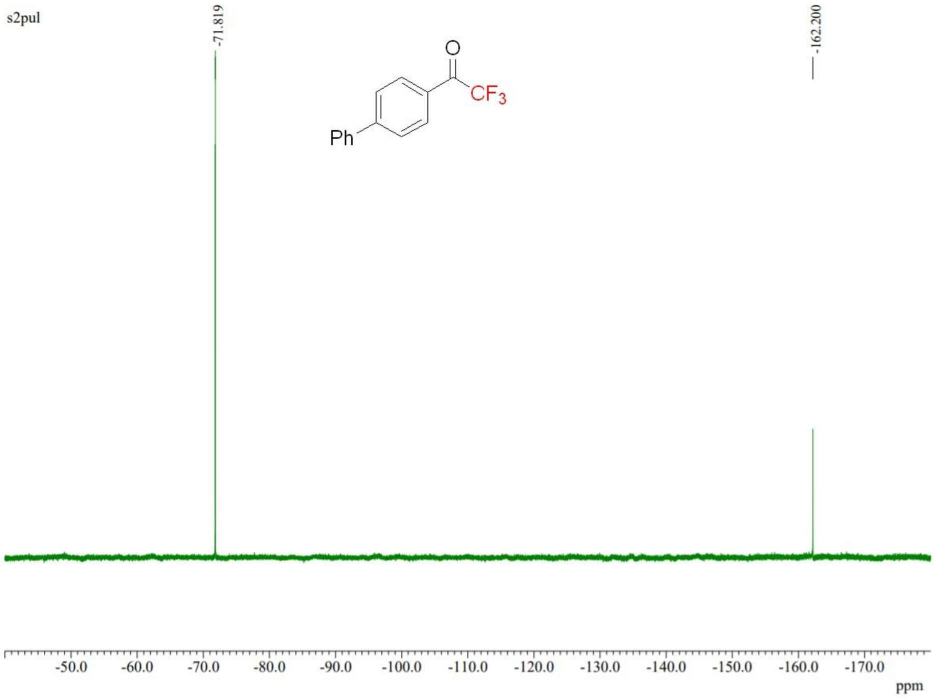

^{1}H NMR (300 MHz, Chloroform-*d*) of **2i**

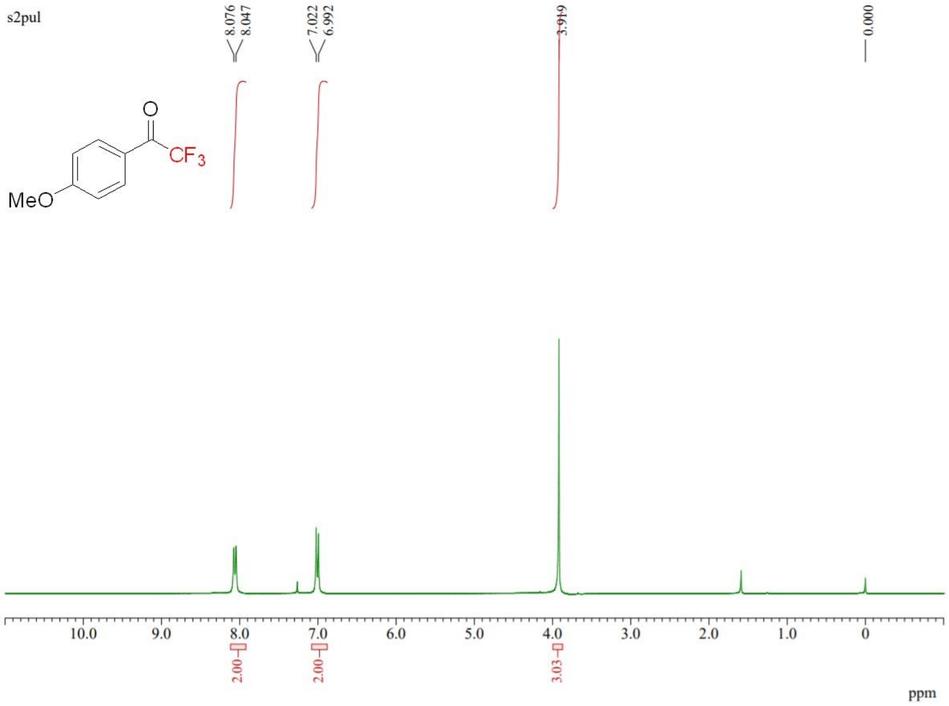

^{19}F NMR (282 MHz, Chloroform-*d*) of **2i**

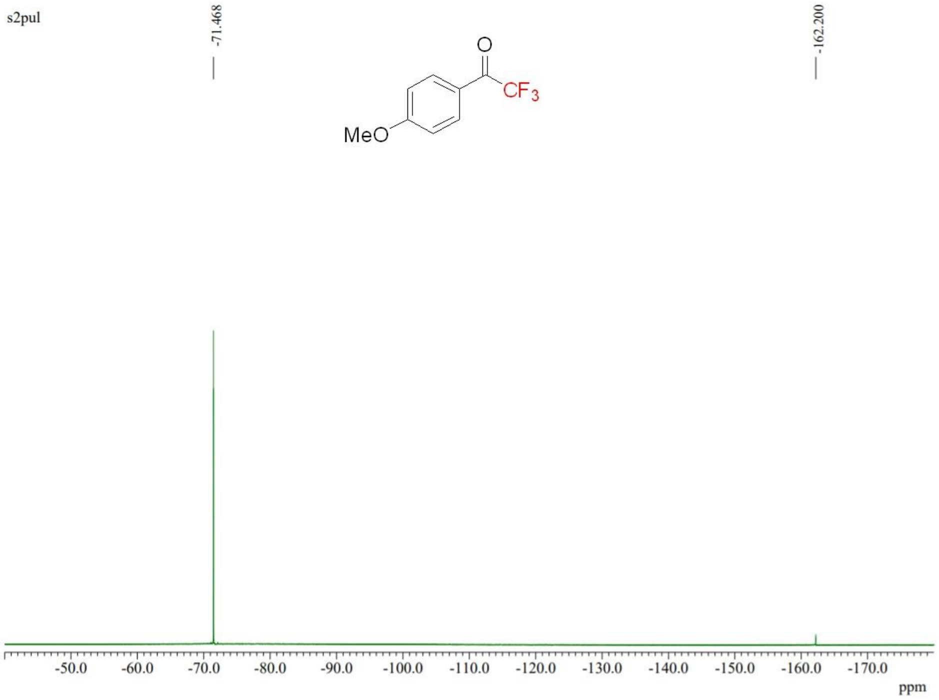

^{1}H NMR (300 MHz, Chloroform-*d*) of **2j**

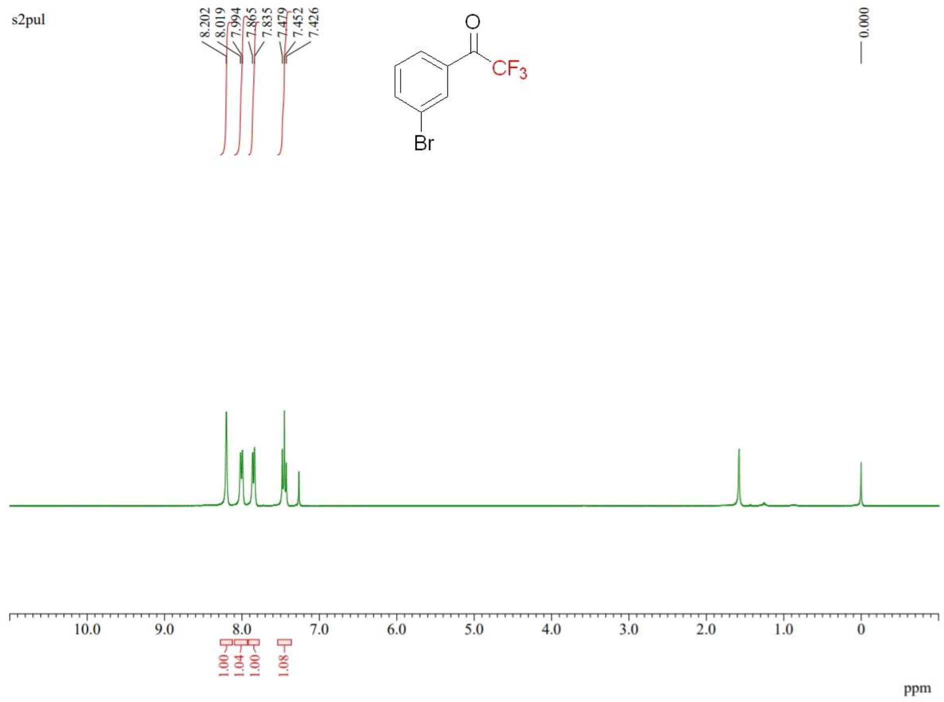

^{19}F NMR (282 MHz, Chloroform-*d*) of **2j**

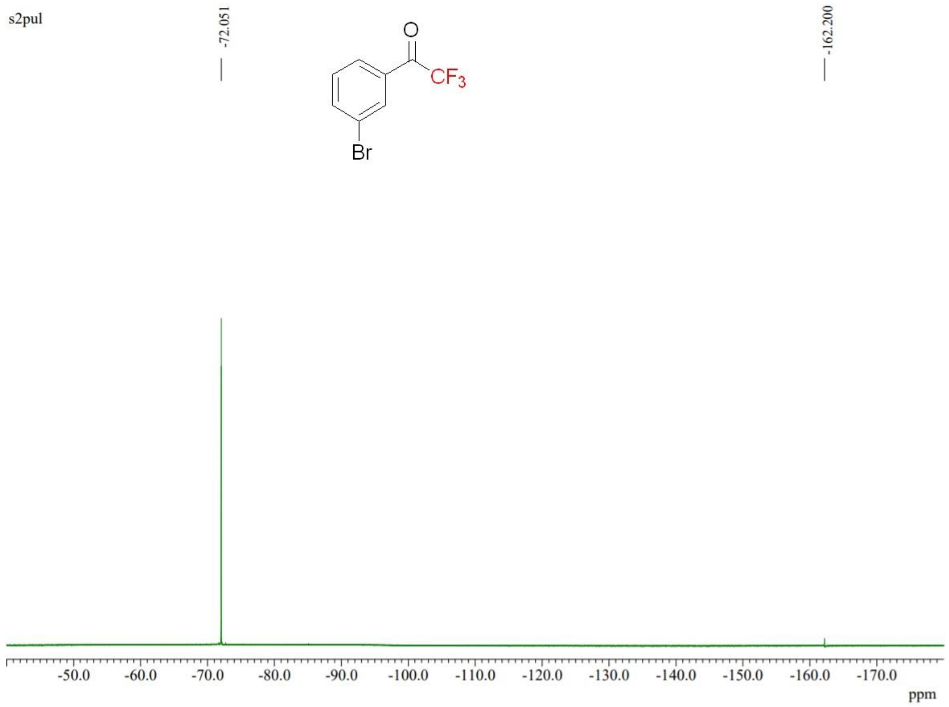

^{1}H NMR (300 MHz, Chloroform-*d*) of **2k**

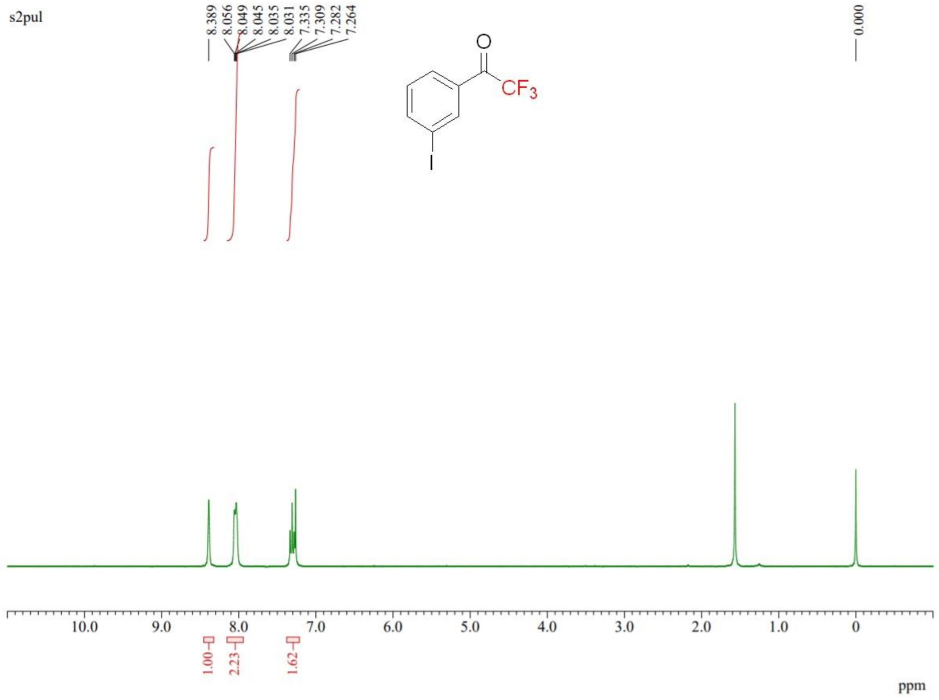

^{19}F NMR (282 MHz, Chloroform-*d*) of **2k**

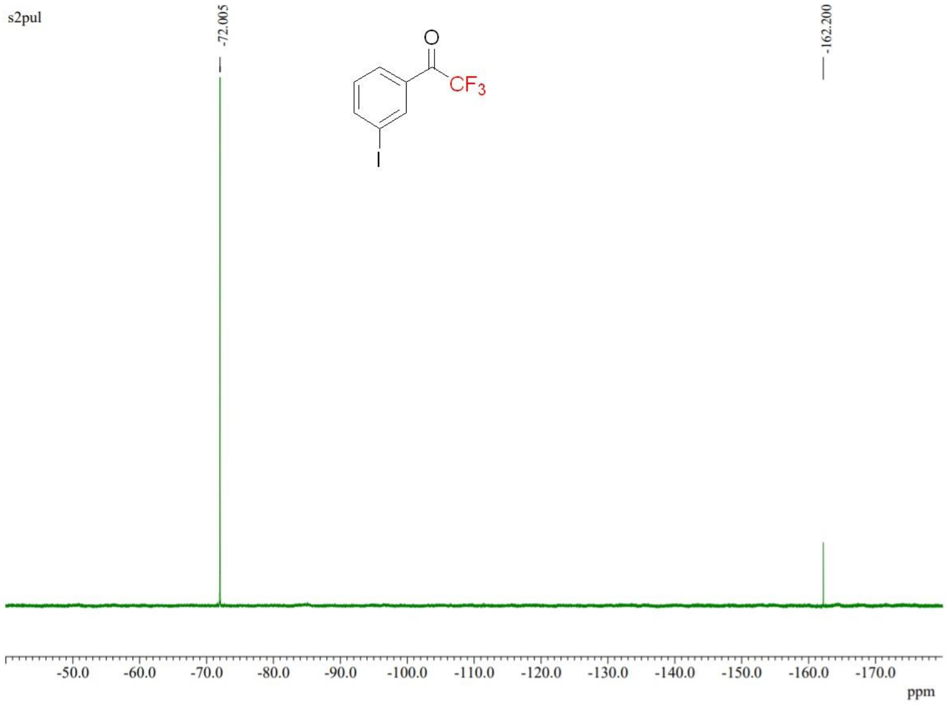

^{1}H NMR (300 MHz, Chloroform-*d*) of **2l**

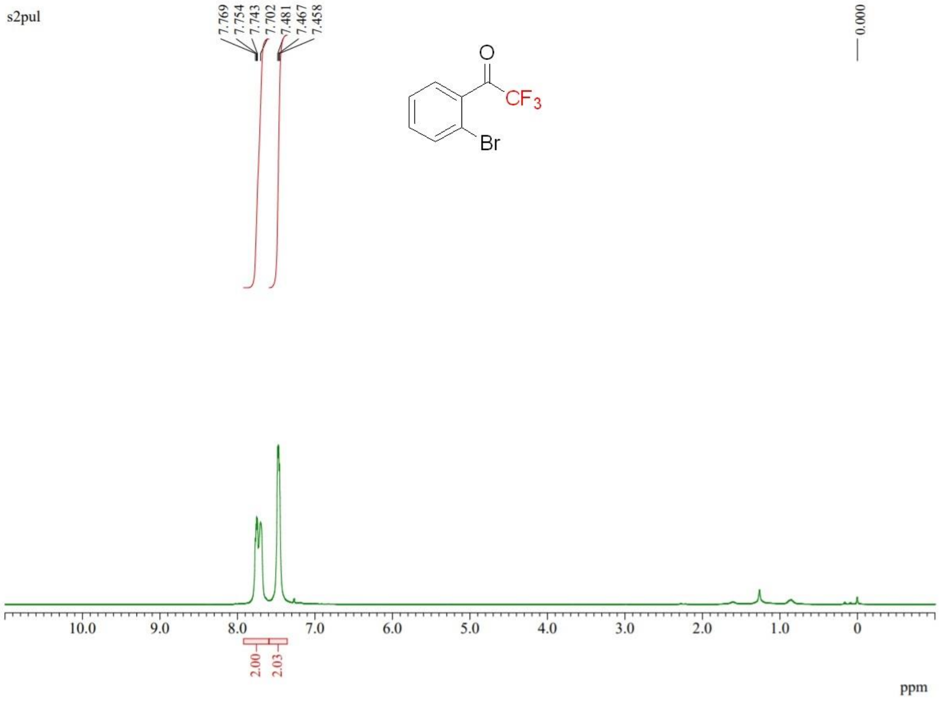

^{19}F NMR (282 MHz, Chloroform-*d*) of **2l**

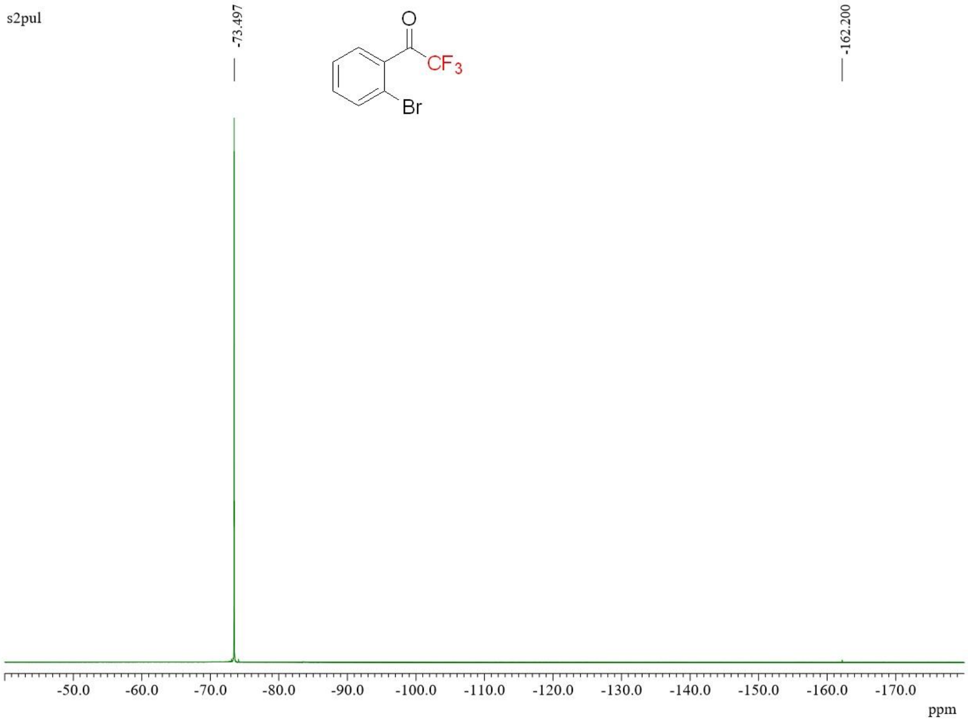

^{1}H NMR (300 MHz, Chloroform-*d*) of **2m**

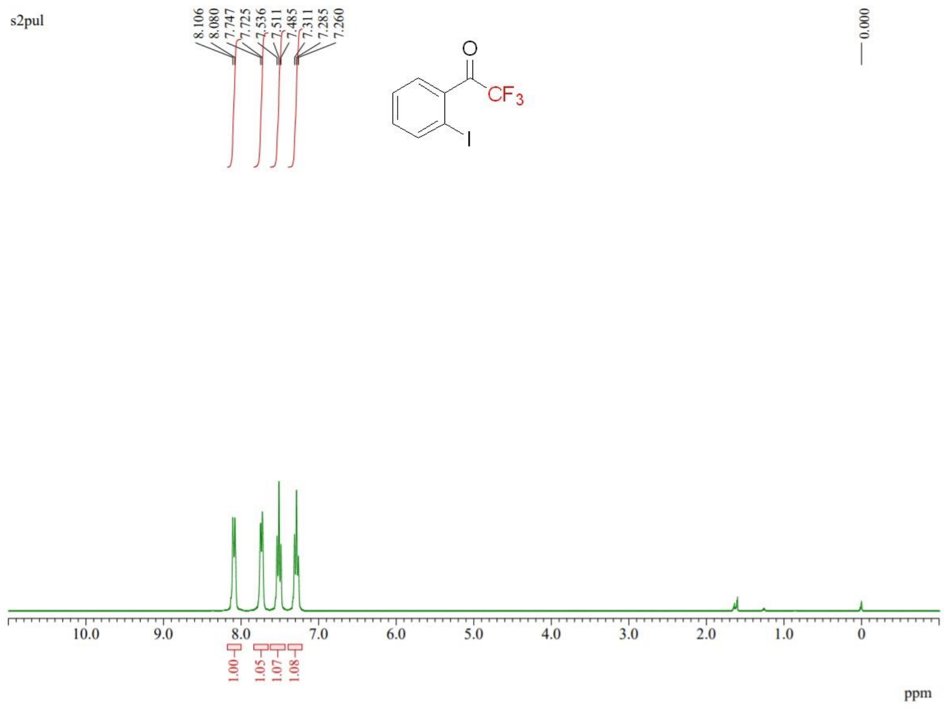

^{19}F NMR (282 MHz, Chloroform-*d*) of **2m**

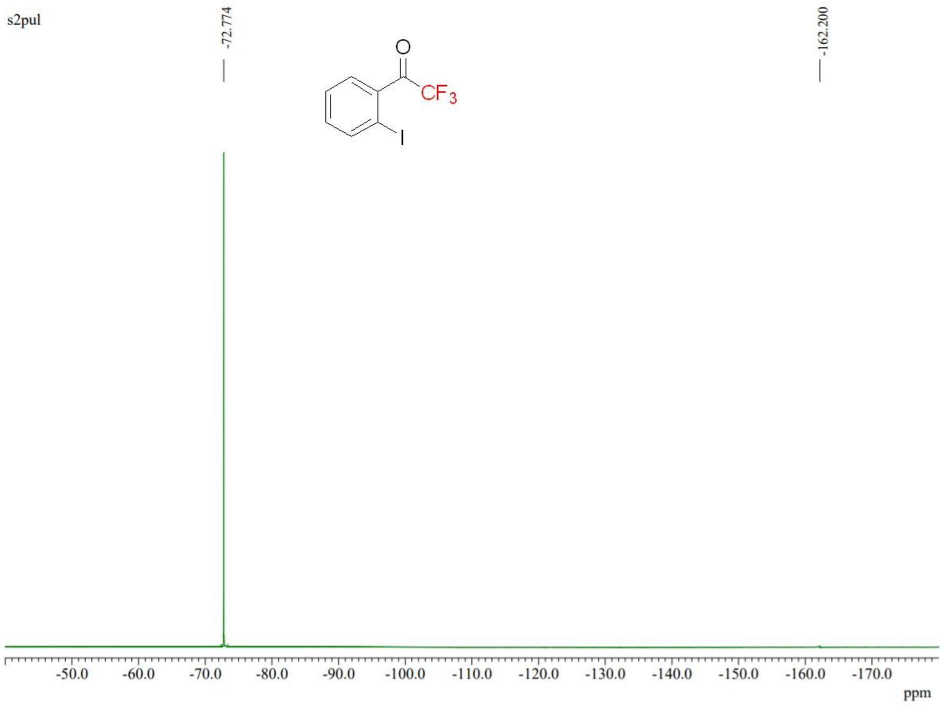

^{13}C {^{1}H} NMR (126 MHz, Chloroform-*d*) of **2m**

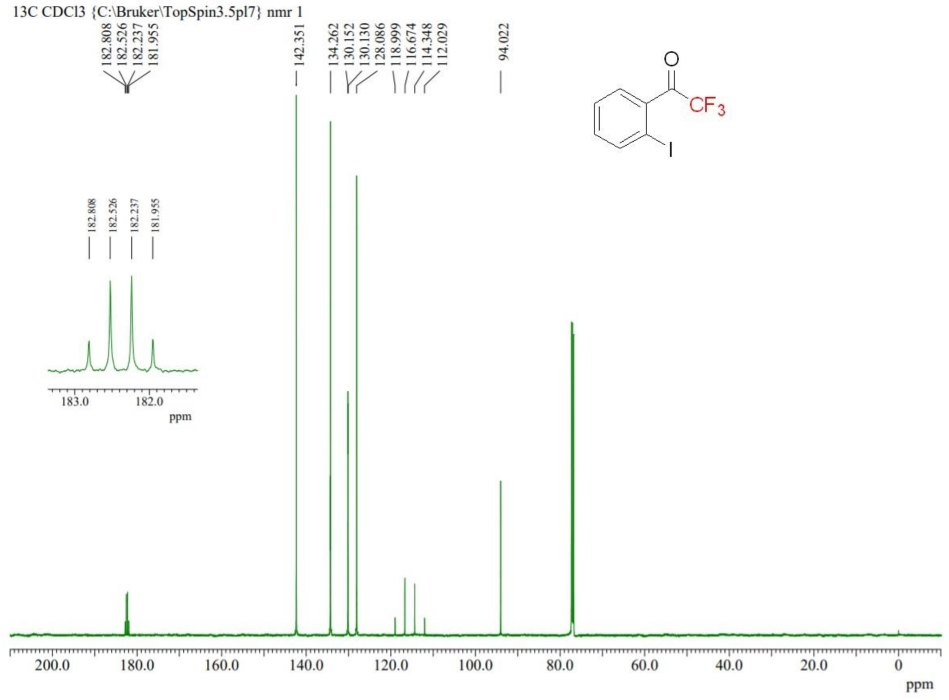

^{1}H NMR (300 MHz, Chloroform-*d*) of **2n**

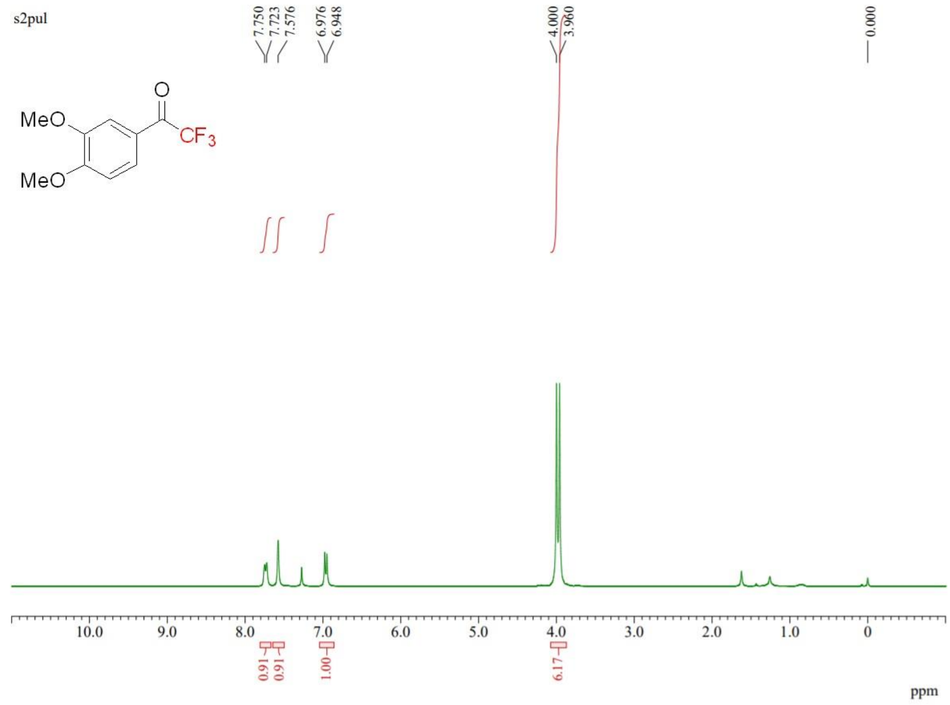

^{19}F NMR (282 MHz, Chloroform-*d*) of **2n**

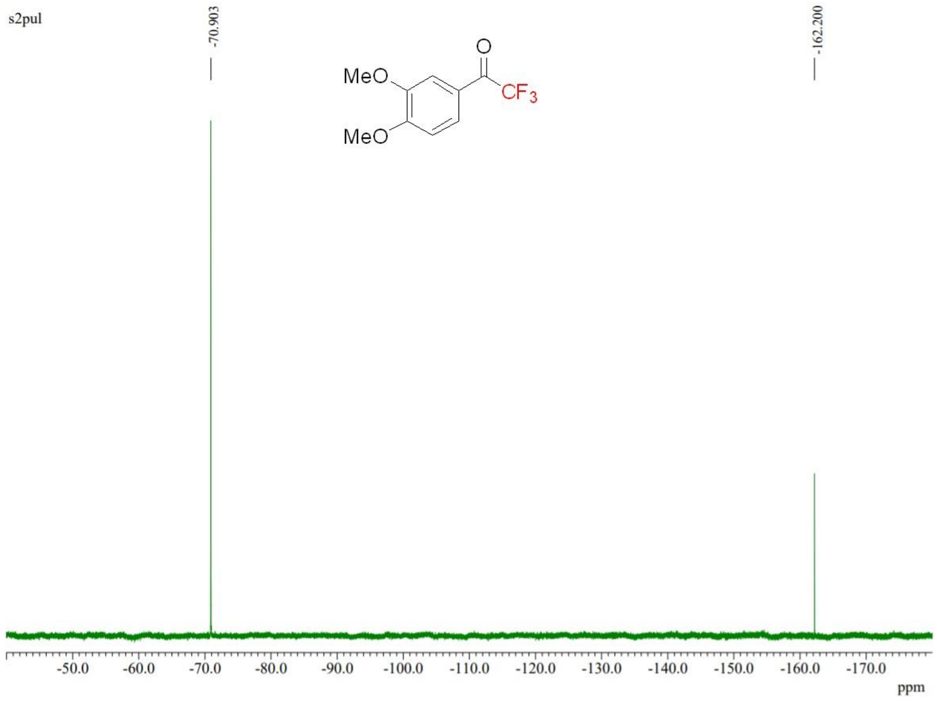

^{1}H NMR (300 MHz, Chloroform-*d*) of **2o**

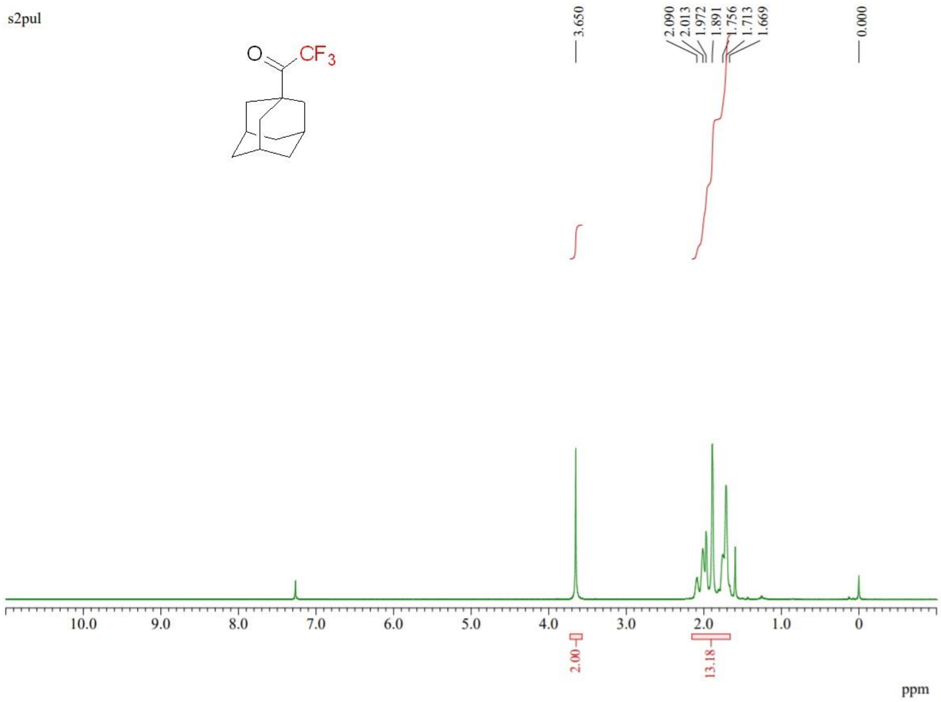

^{19}F NMR (282 MHz, Chloroform-*d*) of **2o**

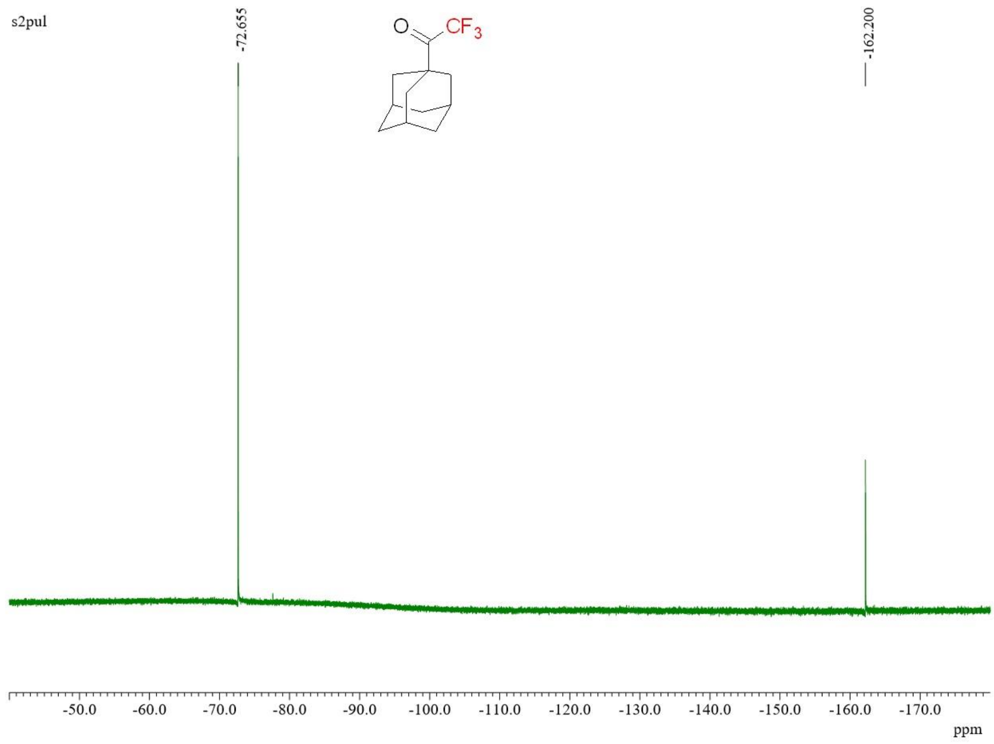

^{1}H NMR (300 MHz, Chloroform-*d*) of **2p**

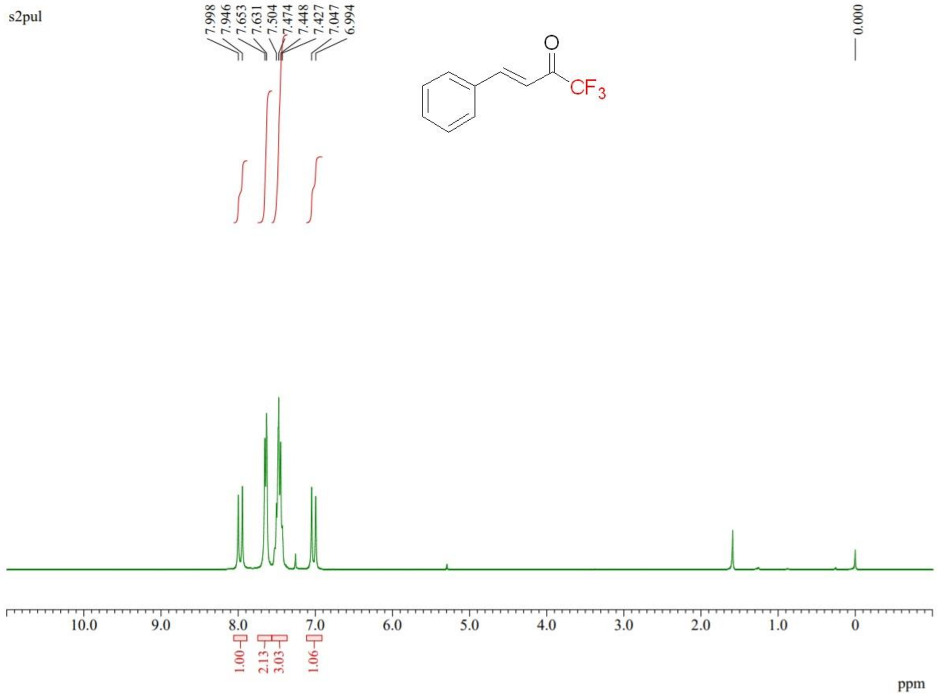

^{19}F NMR (282 MHz, Chloroform-*d*) of **2p**

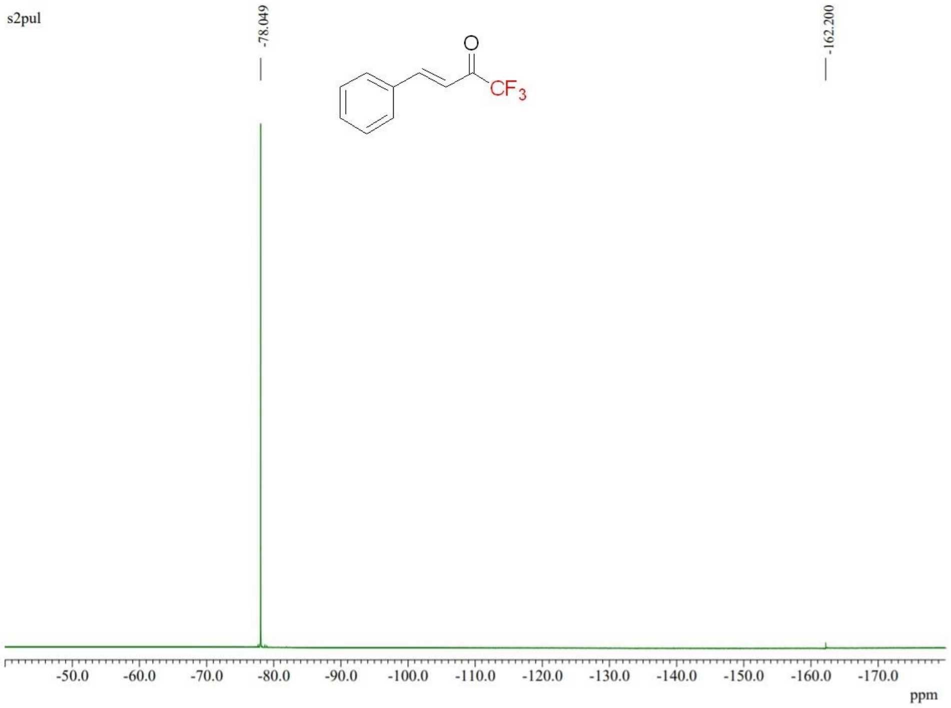

Supplement: File 1 — Optimization of reaction conditions, general procedure and product characterization data. [file Beilstein_J_Org_Chem-17-431-s001.pdf]
